# Spin inversion in graphene spin valves by gate-tunable magnetic proximity effect at one-dimensional contacts

**DOI:** 10.1038/s41467-018-05358-3

**Published:** 2018-07-20

**Authors:** Jinsong Xu, Simranjeet Singh, Jyoti Katoch, Guanzhong Wu, Tiancong Zhu, Igor Žutić, Roland K. Kawakami

**Affiliations:** 1 Department of Physics, The Ohio State University, Columbus, OH 43210 USA; 2 Department of Physics, University at Buffalo, State University of New York, Buffalo, New York 14260 USA

## Abstract

Graphene has remarkable opportunities for spintronics due to its high mobility and long spin diffusion length, especially when encapsulated in hexagonal boron nitride (h-BN). Here, we demonstrate gate-tunable spin transport in such encapsulated graphene-based spin valves with one-dimensional (1D) ferromagnetic edge contacts. An electrostatic backgate tunes the Fermi level of graphene to probe different energy levels of the spin-polarized density of states (DOS) of the 1D ferromagnetic contact, which interact through a magnetic proximity effect (MPE) that induces ferromagnetism in graphene. In contrast to conventional spin valves, where switching between high- and low-resistance configuration requires magnetization reversal by an applied magnetic field or a high-density spin-polarized current, we provide an alternative path with the gate-controlled spin inversion in graphene.

## Abstract (web summary)

Owing to its long spin diffusion length, graphene shows promise for spintronics applications, especially when encapsulated within hexagonal boron nitride. Here, the authors demonstrate gate-tunable spin transport in encapsulated graphene-based spin valves with one-dimensional ferromagnetic edge contacts via magnetic proximity effect.

## Introduction

Spintronics aims for information storage and logic operations by utilizing the electron’s spin degree of freedom in addition to its charge. Beyond the three basic processes of spin injection, spin transport, and spin detection, it is crucial to explore new methods of spin manipulation^1–9^ in order to develop novel architectures for spin-based logic. Graphene has become established as an excellent material for spin transport^10–17^, and there recently have been important advances in the control and modulation of spin transport in graphene spin valves. In graphene with transition metal dichalcogenide (TMD) overlayers, the full modulation of spin currents was demonstrated using electrostatic gates to tune spin absorption into the TMD^6,8^. In graphene constrictions, the spin transport signal was controlled by gate-tunable quantum interference at low temperatures (<~5 K)^3^. In graphene spin valves with h-BN tunnel barriers, inversion of spin signal has been observed as a function of bias current^17^ and h-BN thickness^18^, but gate-dependent inversion of the spin signal was not observed. Alternatively, the MPE in graphene from a ferromagnetic insulator (FI) has been used to fully modulate spin currents by controlling the FI magnetization direction^7,9^. Beyond these experimental advances, Lazić et al. proposed a different type of MPE from simple ferromagnetic metals that can be used to invert the polarization of spin current as a function of electrostatic gate, but the gate-tunable MPE has yet to be realized^19,20^.

In this paper, we experimentally demonstrate the gate-tunable MPE in encapsulated graphene spin valves with 1D ferromagnetic edge contacts and observe an inversion of the non-local spin signal as a function of electrostatic backgate voltage. To investigate the origin of the non-local spin signal inversion, we fabricate hybrid spin valves that employ both 1D contacts and traditional top contacts (2D contacts) for spin injector and detector. By comparing the gate-dependent non-local spin signal for injector-detector combinations consisting of 1D–1D contacts vs. 1D–2D contacts, we are able to establish that the inversion of the non-local spin signal originates from an inversion of the effective spin polarization of the 1D contact as a function of gate voltage. This realizes the prediction by Lazić et al.^20^ that a MPE associated with the spin-dependent DOS of a metallic ferromagnetic electrode could be used to modulate or even invert its effective spin polarization as a function of gate voltage. Unlike 2D contacts where the Fermi level of an adjacent graphene is strongly pinned^21–23^, the Fermi level of graphene near a 1D contact can be tuned efficiently so that different energy levels of the ferromagnet’s spin-polarized DOS can be accessed for gate-tunable MPE. This work provides a new method for manipulating both the magnitude and sign of the spin signal in graphene spin valves, and enables new device architectures for prospective graphene-based spin logic applications.

## Results

### Device structure and charge transport characterization

**Fig. 1:**
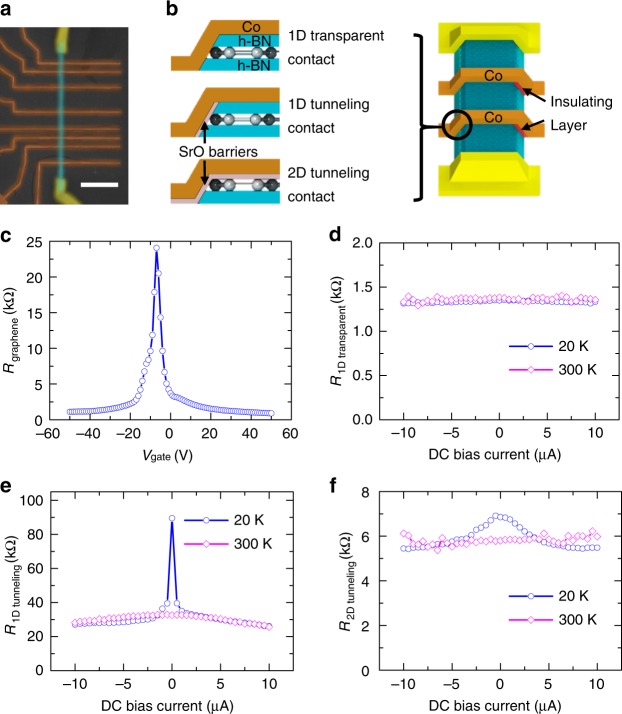
Electrical characterization of graphene and contact resistances. **a** Scanning electron microscope (SEM) image with false color of one of the measured devices (sample I). The scale bar is 10 µm. **b** Schematic of device and three different types of contacts. Co electrodes (brown color) form two 1D contacts with h-BN/graphene/h-BN heterostructure on two sides. The left side is 1D transparent/tunneling contact, and there is 3 nm thick SrO barrier (red color) isolating Co electrode from h-BN/graphene/h-BN on the right edge. So the current is only injecting from the left edge 1D contact. The yellow color is Cr/Au contact. **c** Gate-dependent graphene resistance (sample I). The channel length is 6 µm and the width is 1 µm. **d**–**f** Typical bias dependence of the differential contact resistance for 1D transparent contacts, 1D tunneling contacts, and 2D tunneling contacts, respectively. All measurements are done with 0.1 µA AC excitation current

**Fig. 2:**
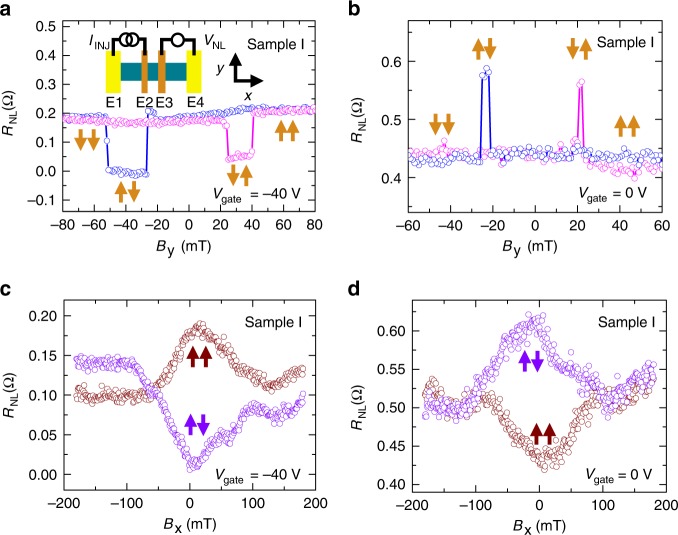
Spin inversion in graphene spin valves with 1D transparent contacts. **a**, **b** Non-local spin signal *R*_NL_ (sample I) for gate voltages of *V*_gate_ = −40 V and *V*_gate_ = 0 V, respectively. The red (blue) curve is for increasing (decreasing) magnetic field. Inset: non-local spin transport geometry. **c**, **d** Non-local Hanle measurement for gate voltages of *V*_gate_ = −40 V and *V*_gate_ = 0 V, respectively. The brown (purple) curve is for parallel (antiparallel) alignment of the injector and detector magnetizations. The lock-in measurements utilize a 5 µA excitation current

Figure 1a shows a scanning electron microscope (SEM) image of one of the measured devices (sample I), and Fig. 1b is a schematic drawing of the device with three types of contacts: 1D transparent contacts, 1D tunneling contacts, or 2D tunneling contacts. The device consists of a h-BN/graphene/h-BN heterostructure on SiO_2_(300 nm)/Si substrate (acting as backgate), contacted by nonmagnetic Cr/Au electrodes at two ends and ferromagnetic Co electrodes in between. Details of device fabrication are discussed in the Supplementary Information, Note 1. Figure 1c shows a typical four-probe resistance of graphene as a function of gate voltage, *V*_gate_ (the channel is 6 µm long and 1 µm wide). This yields a mobility of ~30,000 cm^2^ V^−1^ s^−1^ for holes and ~20,000 cm^2^ V^−1^ s^−1^ for electrons. To characterize the contacts, we perform three-terminal measurement of the differential contact resistance as a function of current bias at 20 K and 300 K (Fig. 1d–f). The 1D transparent contacts exhibit very little variation with either DC bias current or temperature, which is indicative of ohmic conduction (Fig. 1d). For contacts with 0.6 nm SrO barriers, both 1D (Fig. 1e) and 2D (Fig. 1f) contacts show strong bias-dependence of the contact resistance at 20 K, indicating non-ohmic conduction.

### Gate-tunable inversion of the spin valve signal

Next, we investigate spin transport with 1D transparent contacts. All measurements are conducted in the non-local geometry (Fig. 2a inset) with lock-in detection and at 20 K. An injection current, *I*_INJ_ (5 µA rms), is applied between the Co spin injector (E2) and nonmagnetic electrode (E1), and spin transport from E2 to Co spin detector (E3) is measured as a non-local voltage (*V*_NL_) measured across E3 and nonmagnetic electrode (E4). The non-local resistance, *R*_NL_, is defined as *R*_NL_ = *V*_NL_/*I*_INJ_. Figure 2a shows *R*_NL_ as a function of external magnetic field along the Co electrode (*B*_*y*_) at *V*_gate_ = −40 V. The red (blue) circles are data for increasing (decreasing) *B*_y_. At ~20 mT, an abrupt change in *R*_NL_ is due to switching of the Co electrode magnetizations from parallel to antiparallel configuration. With further increasing magnetic field, *R*_NL_ changes back to its original value as the Co electrode magnetizations align parallel. The spin transport is quantified by the change of non-local resistance Δ*R*_NL_ = *R*_NL_ (parallel) − *R*_NL_ (antiparallel), which is ~0.2 Ω. Interestingly, as gate voltage is tuned to 0 V (Fig. 2b), the curves invert and Δ*R*_NL_ becomes negative.

To confirm that the non-local signal originates from spin transport, we perform in-plane non-local Hanle spin precession measurements^24,25^ by applying an in-plane external magnetic field *B*_x_ perpendicular to Co electrodes. The brown (purple) curves in Fig. 2c, d are the in-plane Hanle curves for the parallel (antiparallel) configuration for *V*_gate_ = −40 and 0 V, respectively. This verifies the gate-dependent spin inversion with Δ*R*_NL_ > 0 for *V*_gate_ = −40 V and Δ*R*_NL_ < 0 for *V*_gate_ = 0 V. The extracted spin lifetimes and spin diffusion lengths are up to ~500 ps and ~10 µm (see Supplementary Note 7). In addition, we further verify that these signals do not originate from local Hall effects associated with magnetic fringe fields or tunneling anisotropic magnetoresistance (see Supplementary Notes 8, 9, and 10). On the other hand, in related work^26^ the Co electrodes can be specially designed to generate fringe fields up to 0.6 T and produce measurable Hall signals in the non-local geometry.

**Fig. 3:**
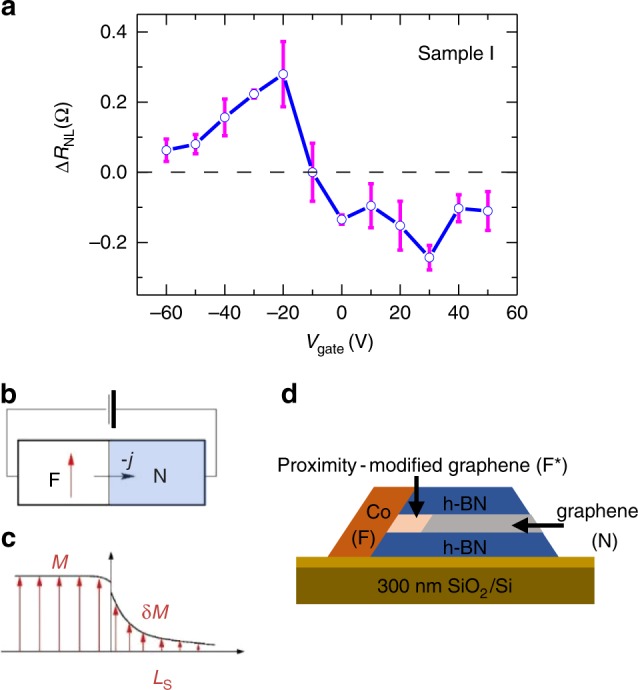
Gate-tunable magnetic proximity effect at 1D transparent contacts. **a** Gate dependence of the non-local spin signal, Δ*R*_NL_ (sample I). The error bars correspond to standard error of mean of the non-local MR with increasing and decreasing magnetic field. **b** Schematic of bulk-like metallic ferromagnet/nonmagnet (F/N) junction (e.g., Co/Cu). **c** Spin accumulation in bulk-like F/N junction. **d** Schematic of the magnetic proximity effect for encapsulated graphene with a transparent 1D edge contact to a ferromagnetic electrode

The detailed gate dependence of the non-local spin signal (Fig. 3a) exhibits a sign reversal at *V*_gate_ ~−10 V. We note that gate-tunable spin inversion has been observed in most of the 1D edge contacted devices (10 out of 11 samples that exhibited spin signals). In addition, we observe substantial sample-to-sample variations including, in some cases, the polarity of the non-local spin signal changing more than once (see Supplementary Note 2).

### Gate-tunable magnetic proximity effect

To understand the origin of the gate-tunable spin inversion, we first rule out quantum interference effects that invert spin signals in small constrictions (few hundred nm) and low temperatures (<5 K)^3^ because our samples are larger (6 µm × 1 µm) and effects persist up to 75 K (see Supplementary Note 3). Instead, the sign change of Δ*R*_NL_ is likely due to the spin-dependent DOS of the 1D ferromagnetic contact, which induces ferromagnetism in the graphene via MPE (see Fig. 3d). In metallic ferromagnet/nonmagnet (F/N) bulk-like junctions, e.g., Co/Cu (Fig. 3b, c), the proximity-induced exchange splitting and magnetization, *M*, decay over a much shorter distance compared to the spin-diffusion length (*L*_S_) in a nonmagnetic material, which determines the spin injection. Consequently, the description of spin injection usually completely neglects the equilibrium proximity effect in nonmagnetic materials. However, in atomically thin materials such as graphene, the magnetic proximity length exceeds their thickness. The wave functions from the metallic ferromagnet penetrate into graphene, directly polarizing its electronic structure at the Fermi level. Thus, describing spin injection for graphene spin valves with 1D edge contacts should include the MPE^20^. Instead of occurring at the F/N interface, the spin injection happens at the F* (proximity-modified graphene)/N interface (Fig. 3d). Previously, evidence for such proximity-modified graphene was observed in vertical Co/graphene/NiFe magnetic tunnel junctions, although the two-terminal geometry does not permit backgate tuning^27^. In addition, X-ray magnetic circular dichroism and angle resolved photoemission experiments directly confirm the magnetic proximity effect for graphene on ferromagnetic metals^28–30^.

To test whether the inversion of Δ*R*_NL_ originates from MPE, we need to demonstrate that the effective spin polarization of the 1D contact exhibits a sign reversal. We accomplish this by designing and testing hybrid spin valve devices with both 1D and 2D contacts. Because Δ*R*_NL_ is roughly proportional to the product of the injector and detector polarization (Δ*R*_NL_ ~ *P*_inj_ *P*_det_)^31,32^, one can investigate spin valves with three ferromagnetic electrodes on the same graphene channel and perform three independent spin transport measurements using the three distinct pairings. Then, one can algebraically extract the effective spin polarization of each contact (see Supplementary Note 4 for details).

**Fig. 4:**
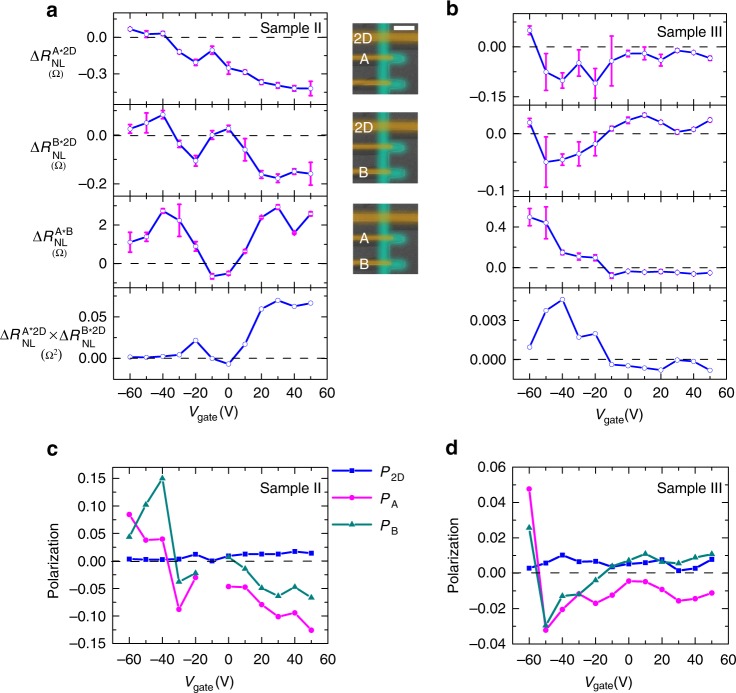
Spin inversion in hybrid graphene spin valves. **a**, **b** Gate dependence of Δ*R*_NL_ for two hybrid devices, sample II and sample III. The spin injector and detector are 1D contact A and 2D contact for the first row, 1D contact B and 2D contact for the second row, 1D contact A and 1D contact B for the third row, respectively, as shown in the central column of SEM images. The scale bar is 2 µm. The last row is the calculated product of the first two rows. **c**, **d** Effective spin polarization of each contact for sample II and sample III. The error bars correspond to standard error of mean of the non-local MR with increasing and decreasing magnetic field

Figure 4a, b show the gate dependence of Δ*R*_NL_ for two hybrid devices (sample II and sample III), each consisting of two 1D transparent contacts (“A” and “B”) and a 2D tunneling contact. The top row is Δ*R*_NL_ measured using 1D contact A and the 2D contact $\left( {{\mathrm{\Delta }}R_{{\mathrm{NL}}}^{{\mathrm{A}} \ast 2{\mathrm{D}}}} \right)$, while the second row is Δ*R*_NL_ measured using 1D contact B and the 2D contact $\left( {{\mathrm{\Delta }}R_{{\mathrm{NL}}}^{{\mathrm{B}} \ast 2{\mathrm{D}}}} \right)$. Here, the observed inversion of spin signal suggests a polarity reversal of the 1D contact because 2D contacts typically show weak gate dependence (see Supplementary Note 5). The third row is Δ*R*_NL_ measured using 1D contact A and 1D contact B $\left( {{\mathrm{\Delta }}R_{{\mathrm{NL}}}^{{\mathrm{A}} \ast {\mathrm{B}}}} \right)$, while the last row $\left( {{\mathrm{\Delta }}R_{{\mathrm{NL}}}^{{\mathrm{A}} \ast 2{\mathrm{D}}} \times {\mathrm{\Delta }}R_{{\mathrm{NL}}}^{{\mathrm{B}} \ast 2{\mathrm{D}}}} \right)$ is the calculated product of the first two rows. By comparing ${\mathrm{\Delta }}R_{{\mathrm{NL}}}^{{\mathrm{A}} \ast {\mathrm{B}}}$with ${\mathrm{\Delta }}R_{{\mathrm{NL}}}^{{\mathrm{A}} \ast 2{\mathrm{D}}} \times {\mathrm{\Delta }}R_{{\mathrm{NL}}}^{{\mathrm{B}} \ast 2{\mathrm{D}}}$, we notice that they are very similar to each other, including both the sign and the trend. Because ${\mathrm{\Delta }}R_{{\mathrm{NL}}}^{{\mathrm{A}} \ast {\mathrm{B}}}\sim P_{\mathrm{A}}P_{\mathrm{B}}$ and ${\mathrm{\Delta }}R_{{\mathrm{NL}}}^{{\mathrm{A}} \ast 2{\mathrm{D}}} \times {\mathrm{\Delta }}R_{{\mathrm{NL}}}^{{\mathrm{B}} \ast 2{\mathrm{D}}}\sim P_{\mathrm{A}}P_{\mathrm{B}}\left( {P_{2{\mathrm{D}}}} \right)^2$, the similarity of the two curves suggests that *P*_2D_ has relative little gate dependence, as expected. For quantitative analysis, we utilize the Takahashi–Maekawa model^31^ of non-local spin transport and solve for the effective spin polarizations *P*_A_, *P*_B_ and *P*_2D_ in terms of the experimental ${\mathrm{\Delta }}R_{{\mathrm{NL}}}^{{\mathrm{A}} \ast 2{\mathrm{D}}}$, ${\mathrm{\Delta }}R_{{\mathrm{NL}}}^{{\mathrm{B}} \ast 2{\mathrm{D}}}$, and ${\mathrm{\Delta }}R_{{\mathrm{NL}}}^{{\mathrm{A}} \ast {\mathrm{B}}}$ data (see Supplementary Note 4 for details). The resulting gate-dependent effective spin polarizations are shown in Fig. 4c, d for samples II and III, respectively. Although the two samples have different gate dependences, two important trends are observed in both samples. First, as expected, the effective polarization of the 2D contact, *P*_2D_, exhibits relatively little variation with gate voltage. Second, the 1D contacts exhibit polarity inversion as a function of gate voltage. Because the gate-dependent inversion of Δ*R*_NL_ occurs due to polarity inversion of the 1D contacts, this strongly supports our hypothesis that the spin inversion originates from gate-tunable MPE.

## Discussion

Theoretically, this strong gate dependence of the effective spin polarization of the 1D contact comes from the spin-dependent DOS of the 1D interface between graphene and Co as a result of the proximity effect. Lazić et al. reported that the band structure of graphene becomes spin-dependent due to proximity effect to ferromagnetic metals^19,20^. As a result, one can tune the polarity of spin polarization at the interface through adjusting the Fermi level in graphene by gate voltage. In conventional graphene spin valves with 2D contacts, the Fermi level of graphene underneath the ferromagnetic electrode is strongly pinned and cannot be tuned effectively by gate voltage. This is supported by first-principles calculations^20,33^, which show that for a 2D contact (e.g., Co/h-BN/graphene), the graphene becomes strongly n-doped: its Dirac cone is shifted ~0.5 eV below the Fermi level. A simple electrostatic model^20^ predicts that the small graphene DOS is required for effective gating. While this could be possible in 2D contacts by shifting the low-DOS region of the Dirac cone close to the Fermi level, it would require a very large gate-induced electric field, more than an order of magnitude larger than ~0.2 V nm^−1^ from our experiments (~60 V voltage applied on 300 nm SiO_2_ + ~20 nm h-BN dielectric material). In contrast, our spin valves with 1D contacts form less aggressive contacts with Co which have a much smaller effective n-doping of graphene and the shift of the Dirac cone below the Fermi level than what is calculated for 2D contacts^20,33^. Consequently, in 1D contacts graphene’s low-DOS region is attained by a much smaller gate voltage, ensuring that different spin-dependent states are readily accessible by gate tuning. Furthermore, complementary studies of nonlocal spin signal in standard graphene spin valves exhibit spin inversion as a function of bias current^17^, and a comparison of different barrier materials (i.e., h-BN, MgO)^34^ provides evidence for a DOS of Co that reverses spin polarization as a function of energy. Thus, the 1D contacts provide the two necessary ingredients for achieving gate-dependent inversion of the magnetic proximity effect^20^, namely: (1) having the Dirac point of graphene in the F contact region to be near the Fermi level, where the low DOS in graphene at the Fermi level enables substantial changes in P, and (2) having a ferromagnetic DOS with reversal of spin polarization as a function of energy.

We further examine the MPE by inserting a 0.6 nm SrO tunnel barrier at the 1D contact and again observe gate-tunable spin inversion (see Supplementary Note 6 for details). This demonstrates that the MPE can extend across a tunnel barrier, as predicted theoretically^20^.

In conclusion, we demonstrate non-local spin transport in h-BN/graphene/h-BN heterostructures with 1D ferromagnetic contacts, and the non-local spin signal can be tuned by gate voltage effectively and even changes polarity. By designing and testing hybrid spin valve devices with both 1D and 2D contacts, we demonstrate this intriguing behavior originates from the gate-tunable MPE at the 1D contact interface. These results pave the way for future development of graphene spintronics[35]. Gate-controlled spin polarity may overcome the usual need for an applied magnetic field and a magnetization reversal to implement the graphene-based spin logic^36^.

## Methods

### Device fabrication

The general procedure for fabricating h-BN/graphene/h-BN heterostructures is shown in Supplementary Figure 1. First, we mount ~2 mm thick polydimethylsiloxane (PDMS) on a glass slide and cover it with a thin film of polycarbonate (PC). This PC/PDMS stamp is used to pick up top the h-BN flake from an SiO_2_/Si substrate. The top h-BN flake is then aligned and brought into contact with graphene on an SiO_2_/Si substrate to pick up graphene with this top h-BN flake. Then the whole stack is aligned and brought into contact with the bottom h-BN flake on an SiO_2_/Si substrate. After contact, the PC film is cut from the glass slide and the entire PC/h-BN/graphene/h-BN combination remains on SiO_2_/Si substrate. The PC film is then dissolved in chloroform. After that, the transferred h-BN/graphene/h-BN heterostructure is cleaned of polymer residue by annealing at 350 °C in ultra-high vacuum (UHV) for 1 h. Then the h-BN/graphene/h-BN graphene heterostructure is patterned by e-beam lithography with PMMA resist and etched by low-power inductively coupled plasma reactive ion etch (ICP-RIE) to get the desired geometry. This process is followed by another annealing step in UHV to remove PMMA residue. Subsequently, we use two steps of e-beam lithography with MMA/PMMA bilayer resist to fabricate electrodes. In the first step, Au electrodes (70 nm) are deposited on the h-BN/graphene/h-BN heterostructure using an e-beam source and a 5 nm Cr underlayer for adhesion. In the second step, Co (60 nm) electrodes are directly deposited in an MBE chamber for one-dimensional (1D) transparent contacts. For tunnel barrier contacts, Co electrodes with SrO tunnel barriers are deposited using angle evaporation with polar angle of 0° for the SrO masking layer (3 nm), 10° for the SrO tunnel barrier (0.6 nm), and 6° for the Co electrode (60 nm). As shown in Supplementary Figure 2, there are a total of four different device geometries: (1) strip shape with 1D contact, (2) Hall bar shape with 1D contact, (3) 2D contact, and (4) 1D combining with 2D contact. For the strip shape device, on the right edge of the heterostructure, a 3 nm SrO barrier is deposited before Co deposition to form insulating layer to block any conduction. For 2D contact and 1D combining with 2D contact device, the top h-BN, before transfer, is etched with several slits which are used for 2D contact deposition. That is, there is no top h-BN in the red color Co electrode region in Supplementary Figure 2(c) and (d).

### Electrical measurements

The measurements are performed in a variable temperature cryostat with vector magnets. The system pressure reaches 1.2 × 10^−7^ mbar at 20 K. The current is applied using a Keithley 6221 current source and the gate voltage is applied using a Keithley 2400 source meter. The voltage signal is detected with a lock-in.

### Data availability

The data that support the findings of this study are available from the corresponding authors on reasonable request.

## Electronic supplementary material

Supplementary Information
