# Supplementary material for: Spin inversion in graphene spin valves by gate-tunable magnetic proximity effect at one-dimensional contacts
Source: Nat Commun. 2018 Jul 20;9:2869. doi: 10.1038/s41467-018-05358-3 (PMC6054683; doi:10.1038/s41467-018-05358-3)
Supplement: Supplementary file 1 — Supplementary Information [file 41467_2018_5358_MOESM1_ESM.pdf]

Supplementary Information for:

Spin inversion in graphene spin valves by gate-tunable magnetic proximity effect at one-dimensional contacts

Jinsong Xu,[1] Simranjeet Singh,[1] Jyoti Katoch,[1] Guanzhong Wu,[1] Tiancong Zhu,[1] Igor Žutić,[2] Roland K. Kawakami[1]

[1]*Department of Physics, The Ohio State University, Columbus, OH 43210, USA*

[2]*Department of Physics, University at Buffalo, State University of New York, Buffalo, New York 14260, USA*

Supplementary Note 1. Device fabrication

The general procedure for fabricating h-BN/graphene/h-BN heterostructures is shown in Supplementary Figure 1. First, we mount ~2 mm thick polydimethylsiloxane (PDMS) on a glass slide and cover it with a thin film of polycarbonate (PC). This PC/PDMS stamp is used to pick up top the h-BN flake from an SiO_2/Si substrate. The top h-BN flake is then aligned and brought into contact with graphene on an SiO_2/Si substrate to pick up graphene with this top h-BN flake. Then the whole stack is aligned and brought into contact with the bottom h-BN flake on an SiO_2/Si substrate. After contact, the PC film is cut from the glass slide and the entire PC/h-BN/graphene/h-BN combination remains on SiO_2/Si substrate. The PC film is then dissolved in chloroform. After that, the transferred h-BN/graphene/h-BN heterostructure is cleaned of polymer residue by annealing at 350 ^{o}C in ultra-high vacuum (UHV) for 1 hour. Then the h-BN/graphene/h-BN graphene heterostructure is patterned by e-beam lithography with PMMA resist and etched by low-power inductively coupled plasma reactive ion etch (ICP-RIE) to get the desired geometry. This process is followed by another annealing step in UHV to remove PMMA residue. Subsequently, we use two steps of e-beam lithography with MMA/PMMA bilayer resist to fabricate electrodes. In the first step, Au electrodes (70 nm) are deposited on the h-BN/graphene/h-BN heterostructure using an e-beam source and a 5 nm Cr underlayer for adhesion. In the second step, Co (60 nm) electrodes are directly deposited in an MBE chamber for one-dimensional (1D) transparent contacts. For tunnel barrier contacts, Co electrodes with SrO tunnel barriers are deposited using angle evaporation with polar angle of 0° for the SrO masking layer (3 nm), 10° for the SrO tunnel barrier (0.6 nm), and 6° for the Co electrode (60 nm). As shown in Supplementary Figure 2, there are a total of four different device geometries: (1) strip shape with 1D contact, (2) Hall bar shape with 1D contact, (3) 2D contact and (4) 1D combining with 2D contact. For the strip shape device, on the right edge of the heterostructure, a 3 nm SrO barrier is deposited before Co deposition to form insulating layer to block any conduction. For 2D contact and 1D combining with 2D contact device, the top h-BN, before

transfer, is etched with several slits which are used for 2D contact deposition. That is, there is no top h-BN in the red color Co electrode region in Supplementary Figure 2 (c) and 2 (d).

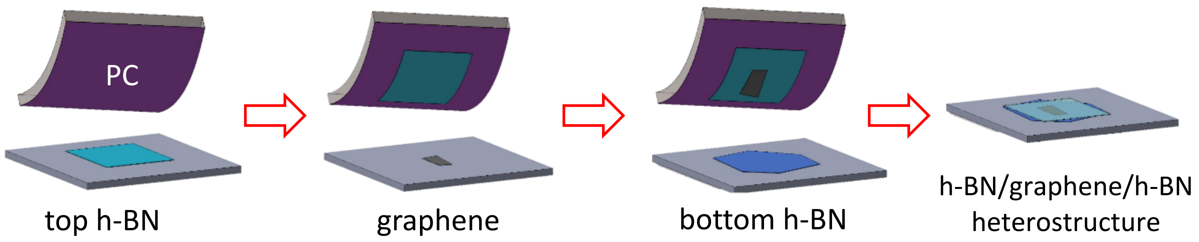

Supplementary Figure 1. Schematic of transfer process.

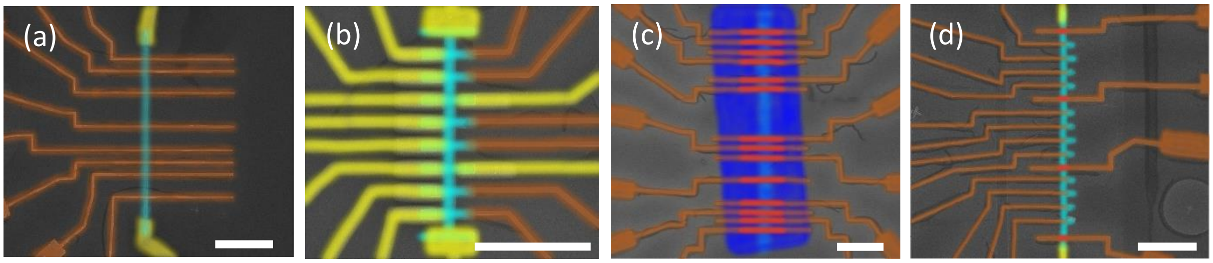

Supplementary Figure 2. Scanning electron microscope images (false color) of four different device geometries. (a) strip shape with 1D contact. (b) Hall bar shape with 1D contact. (c) 2D contact and (d) 1D combining with 2D contact. Light blue is h-BN/graphene/h-BN heterostructure, yellow is Cr/Au electrode, brown is Co electrode and blue is h-BN. The scale is 10 μm.

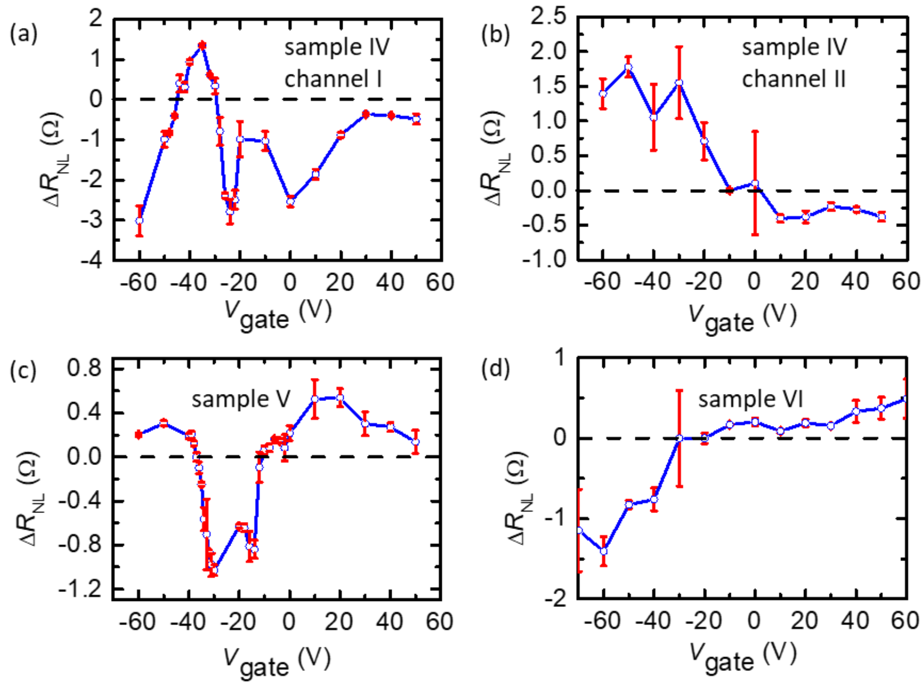

Supplementary Figure 3. Gate dependence of the non-local spin signal ΔR_{NL} of (a) sample IV, channel I (b) sample IV, channel II (c) sample V and (d) sample VI. The error bars correspond to standard error of mean of the non-local MR with increasing and decreasing magnetic field.

Supplementary Note 2. Additional samples

The change in polarity of $\Delta R_{\rm NL}$ as a function of gate voltage is observed in multiple samples with different geometries, which is shown in Supplementary Figure 3. Supplementary Figure 3 (a) and 3 (b) are data from two different pairs of electrodes in sample IV and Supplementary Figure 3 (c) are data from sample V (both sample IV and V have the geometry shown in Supplementary Figure 2 (a)). Supplementary Figure 3 (d) are data from a Hall bar device, sample VI, shown in Supplementary Figure 2 (b).

Supplementary Note 3. Temperature dependence of $\Delta R_{\rm NL}$ for different backgate voltage

Compared to conventional graphene spin valves, there is stronger temperature dependence of $\Delta R_{\rm NL}$ for 1D contact devices. Supplementary Figure 4 shows the temperature dependence of $\Delta R_{\rm NL}$ for different backgate voltage. The negative $\Delta R_{\rm NL}$ is observable up to 75 K and vanishes at 100 K for $V_{\rm gate}$ = 0 V, 20 V and 40 V, while positive $\Delta R_{\rm NL}$ persists to 200 K and disappears at room temperature for $V_{\rm gate}$ = -20 V, -30 V and -50 V.

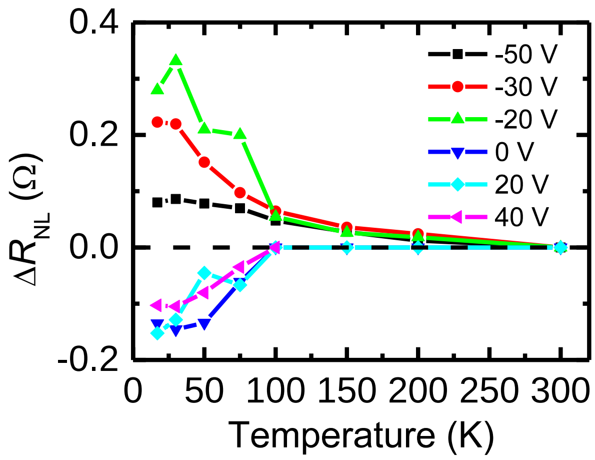

Supplementary Figure 4. Temperature dependence of $\Delta R_{\rm NL}$ for different gate (sample I).

Supplementary Note 4. Extracting the effective spin polarizations of contacts

For quantitative analysis we estimate the polarization of each contact using the model developed by Takahashi and Maekawa[1].

$$\Delta R_{\rm NL} = 2R_{\rm N} e^{-L/\lambda} \frac{\prod_{\rm i=1}^{2}\left(\frac{\frac{P_{\Sigma \rm i} R_{\rm Ci}}{R_{\rm N}}}{1-P_{\Sigma \rm i}{}^2} + \frac{\frac{P_\sigma^{\rm F} R_{\rm F}}{R_{\rm N}}}{1-P_\sigma^{\rm F}{}^2}\right)}{\prod_{\rm i=1}^{2}\left(1+\frac{\frac{2R_{\rm Ci}}{R_{\rm N}}}{1-P_{\Sigma \rm i}{}^2}+\frac{\frac{2R_{\rm F}}{R_{\rm N}}}{1-P_\sigma^{\rm F}{}^2}\right) - e^{-2L/\lambda}}, \qquad (1)$$

where $R_{\rm N} = \frac{\rho_{\rm G}\lambda}{w}$, $R_{\rm F} = \frac{\rho_{\rm F}\lambda_{\rm F}}{A_{\rm J}}$ represents the spin resistance of graphene and Co, λ and $\lambda_{\rm F}$ are spin diffusion length of graphene and Co, L and w is the length and width of graphene (for sample II and III, we use $\lambda =$

7.5 μm, $L_{\mathrm{A*2D}} = 2.5$ μm, $L_{\mathrm{B*2D}} = 5$ μm, $L_{\mathrm{A*B}} = 2.5$ μm and $w = 1$ μm), A_{J} is Co electrode cross section, R_{C} is contact resistance, P_{σ}^{F} is Co spin polarization and P_{Σ} is contact spin polarization, which we consider to be gate-dependent. Because in our samples P_{Σ} is usually less than 0.1 and R_{F} is also much smaller than R_{C}, equation (1) can be simplified to

$$\Delta R_{\mathrm{NL}} = P_{\Sigma 1} P_{\Sigma 2}\, f_{12}(R_{\mathrm{C1}}, R_{\mathrm{C2}}, R_{\mathrm{N}}, L, \lambda), \tag{2}$$

where
$$f_{12}(R_{\mathrm{C1}}, R_{\mathrm{C2}}, R_{\mathrm{N}}, L, \lambda) = \frac{2R_{\mathrm{N}} e^{-L/\lambda} \frac{R_{\mathrm{C1}}}{R_{\mathrm{N}}} \frac{R_{\mathrm{C2}}}{R_{\mathrm{N}}}}{(1+\frac{2R_{\mathrm{C1}}}{R_{\mathrm{N}}})(1+\frac{2R_{\mathrm{C2}}}{R_{\mathrm{N}}}) - e^{-2L/\lambda}}. \tag{3}$$

From equation (2) we can see that the spin signal is the product of spin polarization of spin injector and detector multiplied by a factor $f_{12}(R_{\mathrm{C1}}, R_{\mathrm{C2}}, R_{\mathrm{N}}, L, \lambda)$ determined by R_{C}, R_{N}, L and λ. Since the polarization of 2D contact does not change its polarity with gate voltage, to adopt a convention that its spin polarization is positive (i.e. we can only determine relative polarizations, so we must adopt a sign convention). Then we have

$$\begin{cases} P_{\mathrm{2D}} = \sqrt{\dfrac{\Delta R_{\mathrm{NL}}^{\mathrm{A*2D}} \Delta R_{\mathrm{NL}}^{\mathrm{B*2D}}}{\Delta R_{\mathrm{NL}}^{\mathrm{A*B}}} \dfrac{f_{\mathrm{A*B}}}{f_{\mathrm{A*2D}} f_{\mathrm{B*2D}}}} \\ P_{\mathrm{A}} = \dfrac{\Delta R_{\mathrm{NL}}^{\mathrm{A*2D}}}{f_{\mathrm{A*2D}}} \dfrac{1}{P_{\mathrm{2D}}} \\ P_{\mathrm{B}} = \dfrac{\Delta R_{\mathrm{NL}}^{\mathrm{B*2D}}}{f_{\mathrm{B*2D}}} \dfrac{1}{P_{\mathrm{2D}}} \end{cases} \tag{4}$$

Based on the three measured gate dependent non-local spin signal $\Delta R_{\mathrm{NL}}^{\mathrm{A*2D}}$, $\Delta R_{\mathrm{NL}}^{\mathrm{B*2D}}$ and $\Delta R_{\mathrm{NL}}^{\mathrm{A*B}}$, the contact resistance and graphene resistance at each gate voltage, we calculate the effective spin polarization of each contact using equation (4). The extracted effective spin polarization of each contact for sample II and III are shown in main text Figure 4 (c) and 4 (d).

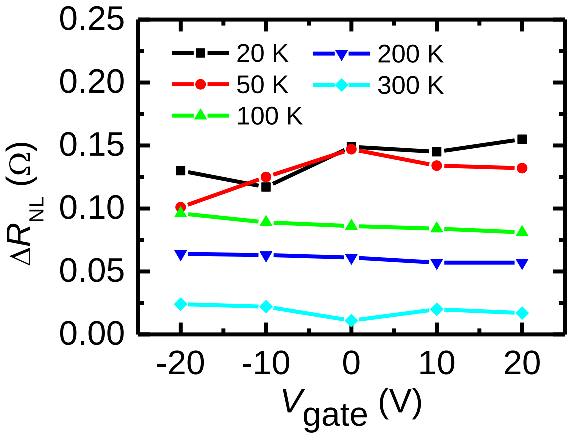

Supplementary Figure 5. Backgate dependence of encapsulated spin valve device with 2D contacts at different temperature (sample VII).

Supplementary Note 5. Backgate dependence of encapsulated spin valve devices with 2D tunneling contacts

Supplementary Figure 5 shows the backgate dependence of the non-local spin signal ΔR_{NL} for an encapsulated spin valve device with 2D tunneling contacts (shown in Supplementary Figure 2 (c)). The device exhibits weak backgate dependence of ΔR_{NL} from 20 K up to room temperature.

Supplementary Note 6. Gate tunable proximity effect with 1D tunneling contact

Our results also suggest that the tunable magnetic proximity effect can exist in the presence of 1D contacts with a tunnel barrier. We measured h-BN/graphene/h-BN 1D contact devices with 0.6 nm SrO tunneling barriers. Supplementary Figures 6 (a) and 6 (b) are the non-local magnetoresistance (MR) curves at V_{gate} = - 40 V and V_{gate} = 40 V. It is clear that the polarity of ΔR_{NL} still changes, even with 0.6 nm SrO tunneling barriers. This agrees with the theory prediction that the magnetic proximity effect can extend across a tunnel barrier[2].

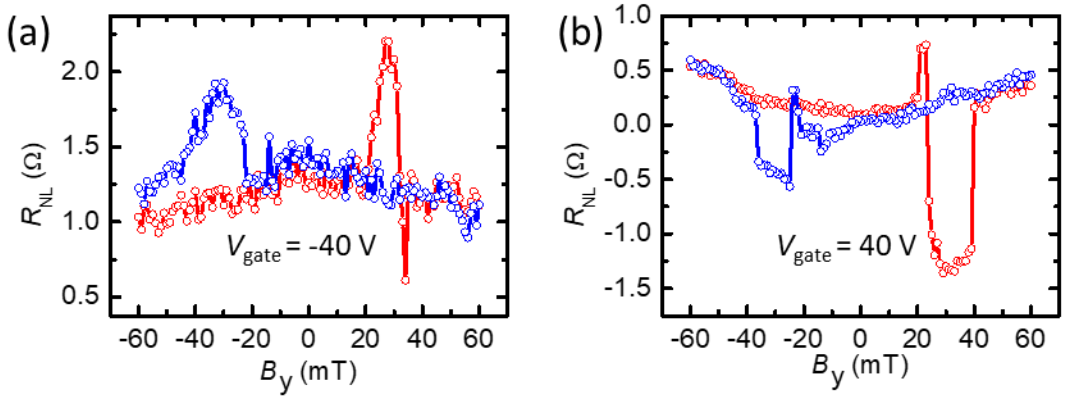

Supplementary Figure 6. Non-local MR curves for graphene spin valve device with 1D tunneling contact. (a) V_{gate} = -40 V and (b) V_{gate} = 40 V. The red (blue) curve is for increasing (decreasing) magnetic field (sample VIII).

Supplementary Note 7. Spin lifetime and diffusion length

To extract spin lifetime and diffusion length, we perform the analysis on the raw data as well as symmetrized data because the curves show significant asymmetry as a function of Bx. We speculate this asymmetry could be due to the non-collinearity between the spin orientation of the 1D interfaces at spin injector and detector. Supplementary Figures 7 (a) and 7 (b) show the raw and symmetrized data for V_{gate} = -40 V, respectively. For raw data in Supplementary Figure 7 (a), we fitted with both standard model[3] (red curve) and modified model considering asymmetric component (blue curve). The origin of the asymmetry is not known but one possibility is the presence of a relative angle between the directions of the effective polarizations of the injector and detector. The extracted spin lifetime and diffusion length

are 252 ± 62 ps (208 ± 12 ps for asymmetric fitting) and 8.6 ± 1.6 µm (7.7 ± 0.4 µm for asymmetric fitting). The extracted spin lifetime and diffusion length are 252 ± 12 ps and 8.6 ± 0.3 µm for symmetrized data in Supplementary Figure 7 (b). Supplementary Figures 7 (c) and 7 (d) show the raw and symmetrized data for V_{gate} = 0 V, respectively. The extracted spin lifetime and diffusion length are 515 ± 122 ps (515 ± 87 ps for symmetrized) and 10.1 ± 1.3 µm (10.0 ± 1.0 µm for symmetrized).

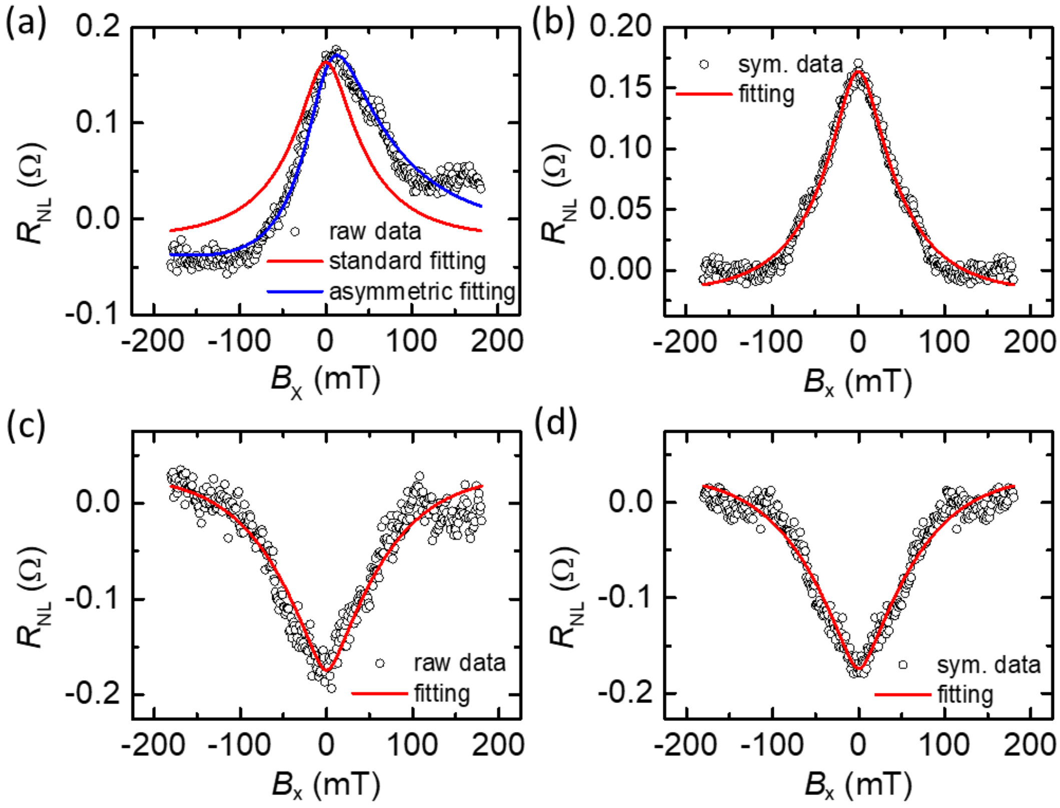

Supplementary Figure 7. Spin lifetime and diffusion length of Sample I shown in main text. Hanle fitting for data shown in main text Figure 2 (c): (a) raw data fitted with standard model (red curve) and modified model considering asymmetric component (blue curve) and (b) symmetrized data fitted with standard model. Hanle fitting for data shown in main text Figure 2 (d): (c) raw data and (d) symmetrized data fitted with standard model.

Supplementary Note 8. Comparison of local Hall effect and our spin transport

One concern is the local Hall effect from the fringe fields of Co electrodes, which may cause some artifacts. Recently published work of B. Karpiak *et al.*[4], suggests that in 1D edge ferromagnet/graphene contacts using a geometry and materials similar to ours, the observed results are dominated by such magnetic stray fields, not the magnetic proximity effects. To compare local Hall effect from fringe fields and our spin transport results, we fabricated a device (sample IX) with the geometry used in B. Karpiak *et*

al. to purposely generate magnetic fringe fields and local Hall effects. Here, the Co electrodes terminate on top of the graphene channel instead of crossing the entire graphene, as shown in Supplementary Figure 8 (a). Supplementary Figure 8 (b) is the gate dependent graphene channel resistance, of which the Dirac point is around V_{gate} = -35 V. Supplementary Figure 8 (c) is the Hall resistance R_{xy} as a function of magnetic field B_z at V_{gate} = -10 V. The slope $d(R_{xy})/d(B_z) = 1/qe$ of this curve is the Hall coefficient R_H, which is summarized in Supplementary Figure 8 (d) for different gate voltage. The Hall coefficient changes sign at Dirac point. Supplementary Figure 8 (e) shows magnetic field B_y dependent non-local resistance R_{NL} at V_{gate} = -40 V and it has a square shape 'hysteresis loop' which is due to the switch of fringe fields from a single Co electrode, while our spin valves data (main text Figure 2 and Supplementary Figure 6) have two jumps due to spin transport from magnetization switches of two Co electrodes. Supplementary Figure 8 (f) shows magnetic field B_y dependent non-local resistance R_{NL} at V_{gate} = -20 V, which has opposite sign to R_{NL} at V_{gate} = -40 V. This is expected from local Hall effect. Supplementary Figure 8 (g) summarizes the change of non-local resistance ΔR_{NL} as a function of gate voltage (red dots). It has the same trend to the Hall coefficient R_H (blue curve). This strongly indicates the signal ΔR_{NL} is due to local Hall effect from the fringe fields of a single Co electrode. In contrast, our spin valve devices have totally different gate dependence. Supplementary Figure 9 is the gate dependent graphene resistance for different samples. Different channels on the same sample have the same Dirac point (Supplementary Figure 9 (a), 9 (b), 9 (c) and (d)), indicating the samples are uniform. However, the gate dependence of ΔR_{NL} for different spin transport channels on the same sample (main text Figure 4 (a) and 4 (b), Supplementary Figure 3 (a) and 3 (b)) is different and ΔR_{NL} for different channels can have different signs at the same gate voltage, i.e. the same carrier type and density because the sample is uniform. And the sign change of ΔR_{NL} in some samples can even happen more than once (Supplementary Figure 3 (a) and 3 (c)). Furthermore, the voltage signal from fringe fields should have a linear dependence on current, i.e. the non-local resistance signal ΔR_{NL} should be current independent as shown in Supplementary Figure 8 (h), which is very different from our spin valve data as shown in Supplementary Figure 10. In addition, while the spin signal ΔR_{NL} from MPE has a strong temperature dependence (Supplementary Figure 4), ΔR_{NL} due to the local Hall effect from the fringe fields of Co electrode has weak temperature dependence and is observed up to room temperature, as shown in Supplementary Figure 11. All these difference between local Hall effect from fringe fields and our spin valves data strongly indicate our spin valves signal is not from local Hall effect from fringe fields, but from spin transport.

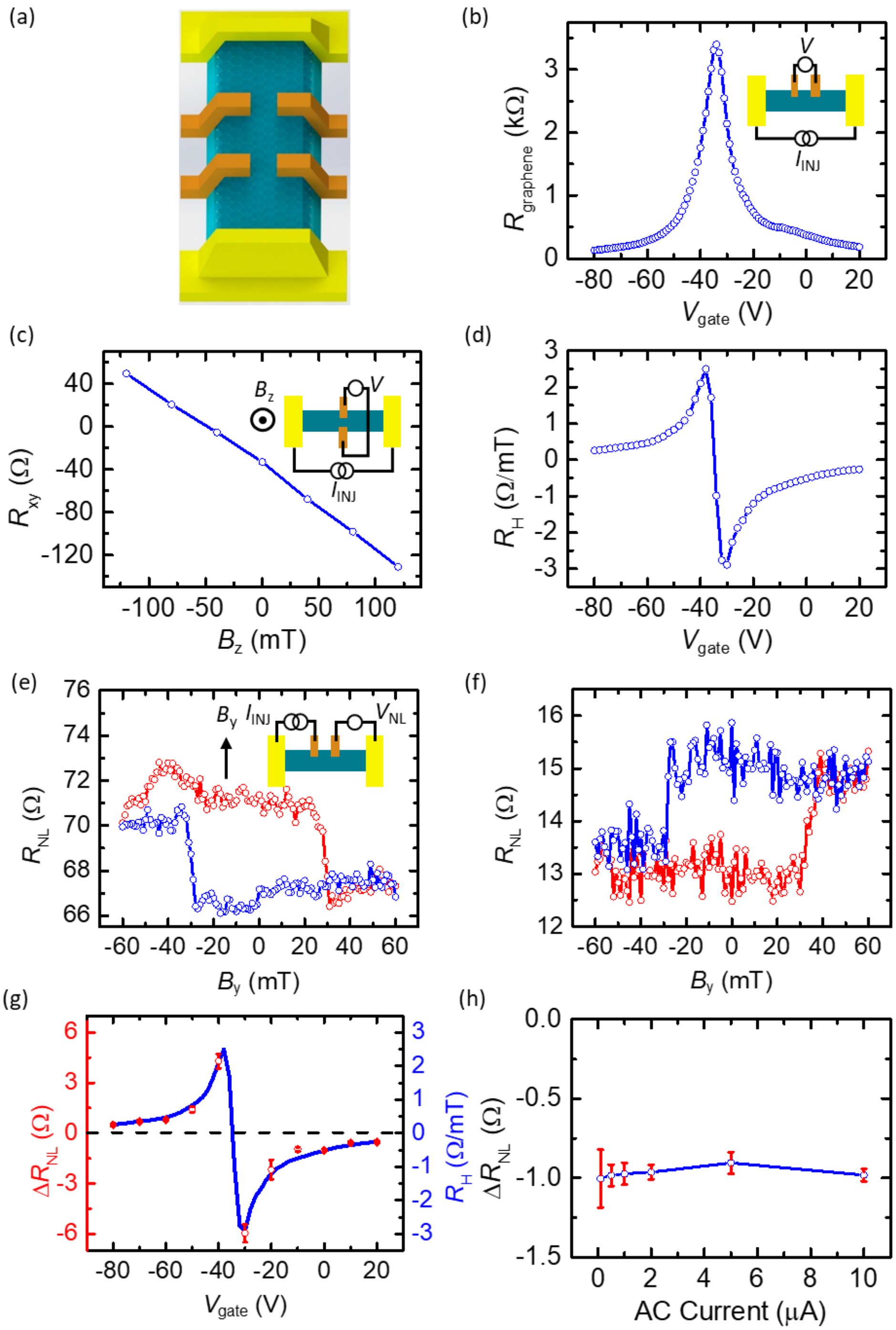

Supplementary Figure 8. Low temperature (T = 20 K) transport measurement on sample IX with fringe fields from the end of Co electrodes. (a) schematic drawing of sample IX, with Co electrodes end on graphene channel generating fringe fields. (b) gate dependent graphene channel resistance. (c) Hall resistance R_{xy} as a function of magnetic field B_z at V_{gate} = -10 V. (d) gate dependent Hall coefficient R_H. (e) and (f), R_{NL} as a function of magnetic field B_y at V_{gate} = -40 V and -20V. The red (blue) curve is for increasing (decreasing) magnetic field. (g) gate dependent ΔR_{NL} (red dots) compared to Hall coefficient R_H (blue curve). (h), ΔR_{NL} as a function of AC current for sample IX. The insets in panels (b), (c) and (e) are measurement diagrams.

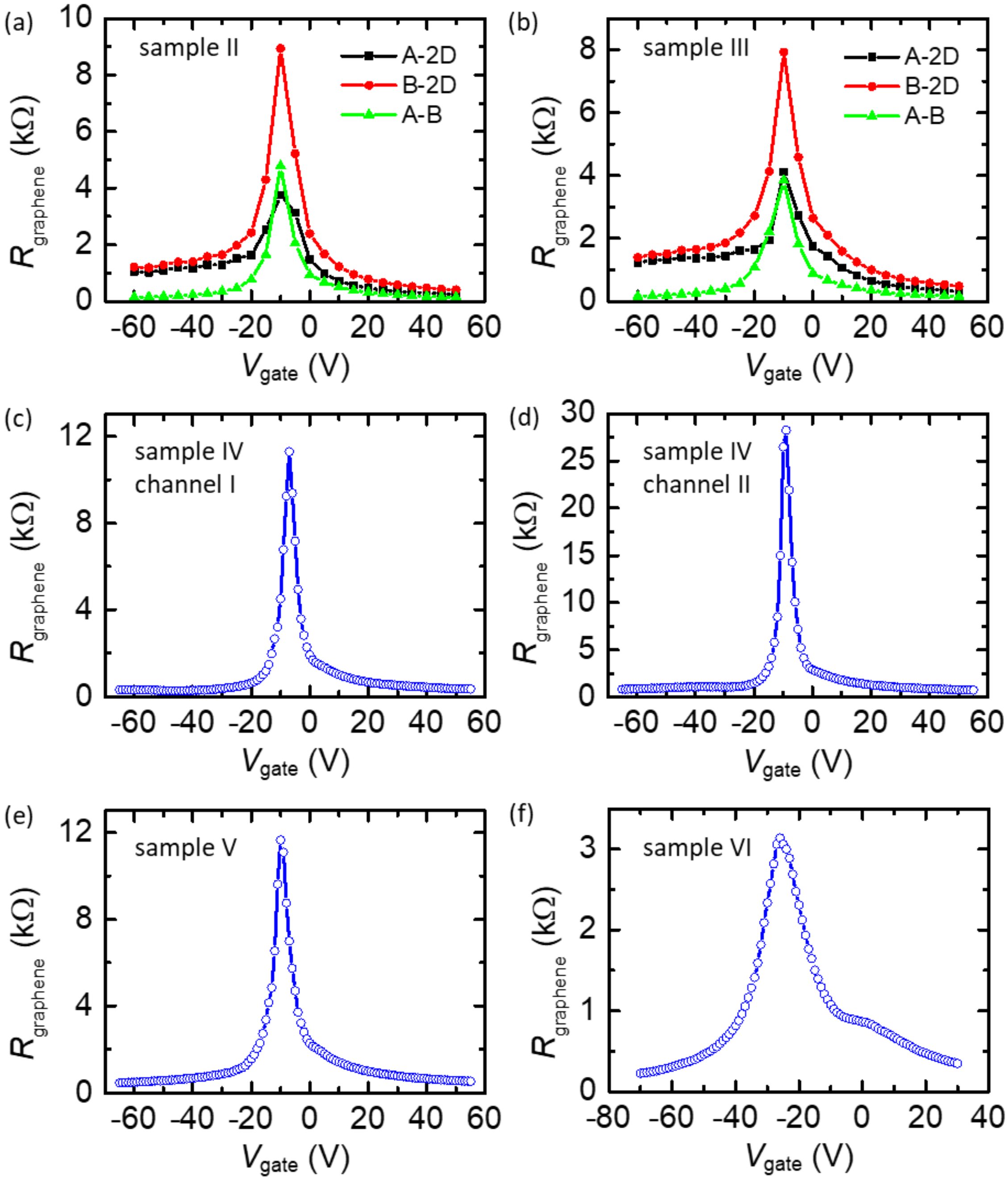

Supplementary Figure 9. Gate dependent graphene channel resistance for (a) sample II (b) sample III (c) sample IV, channel I (d) sample IV, channel II (e) sample V and (f) sample VI.

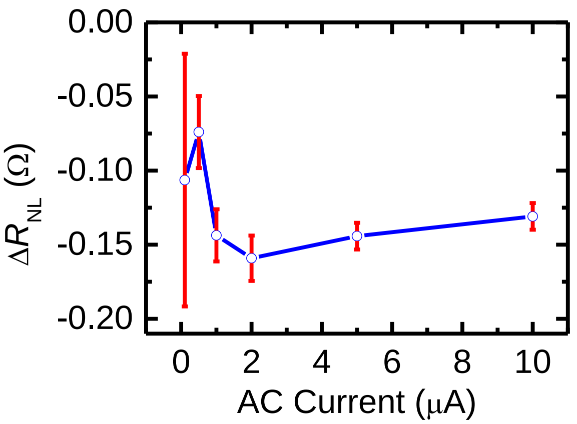

Supplementary Figure 10. AC current dependent non-local resistance ΔR_{NL} from graphene spin valves sample I.

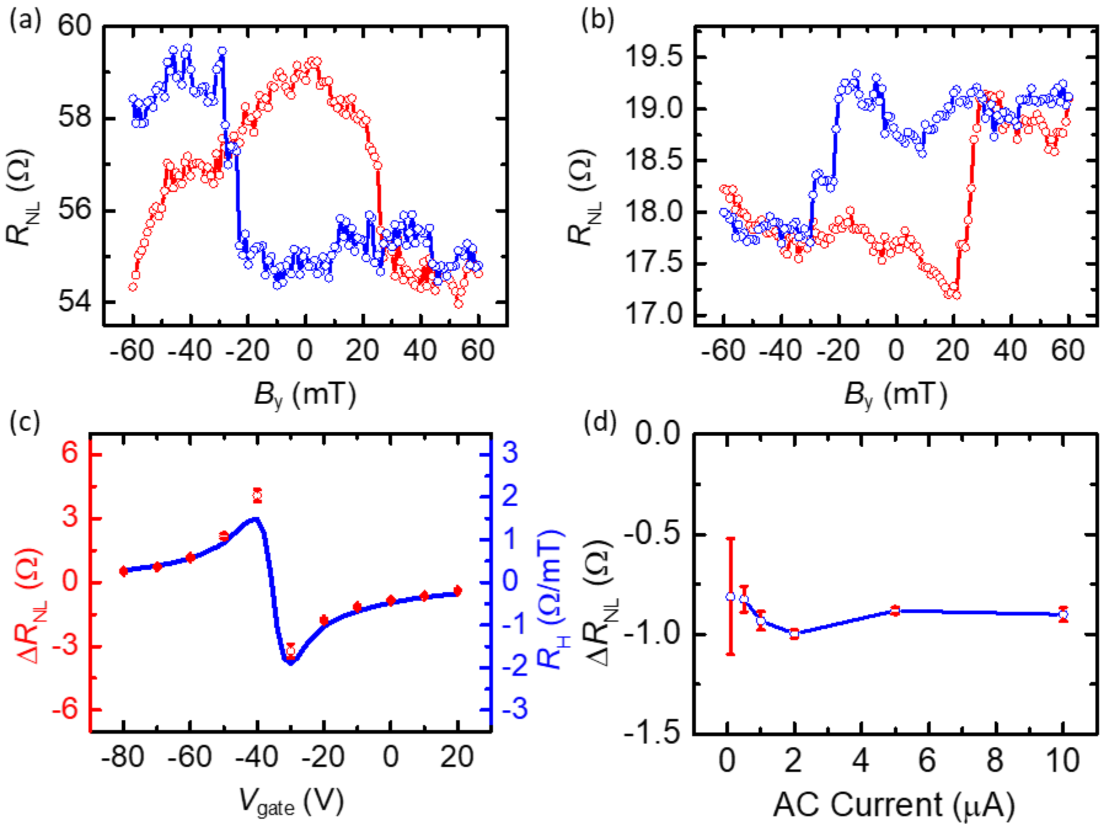

Supplementary Figure 11. Room temperature (T = 300 K) transport measurement on sample IX with fringe fields from the end of Co electrodes. (a) and (b), R_{NL} as a function of magnetic field B_y at V_{gate} = -40 V and -20V. The red (blue) curve is for increasing (decreasing) magnetic field. (c) gate dependent ΔR_{NL} (red dots) compared to Hall co-efficient R_H (blue curve). (d) ΔR_{NL} as a function of AC current for sample IX.

Supplementary Note 9. Tunneling anisotropic magnetoresistance

Another concern is tunneling anisotropic magnetoresistance (TAMR) though one would not expect this effect to be substantial because it requires strong spin-orbit coupling. To rule out this possible artifact,

we measured TAMR effect on the sample device presented in the main text. As shown in Supplementary Figure 12, there is no observable TAMR signal, which rules out TAMR as the origin of the observed spin signal in the main text.

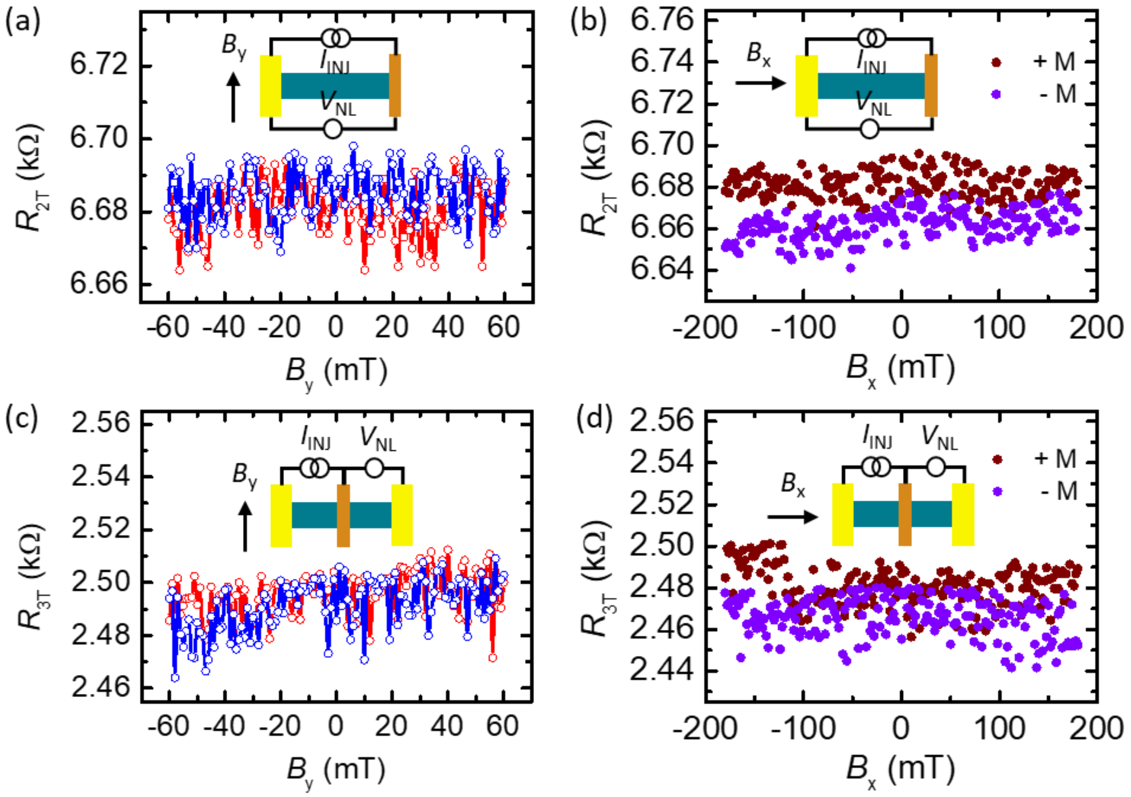

Supplementary Figure 12. TAMR effect measurement at T = 20 K and V_{gate} = 20 V. (a) and (b) are two terminal measurement, (c) and (d) are three terminal measurement. In (a) and (c) the red (blue) curve is for increasing (decreasing) magnetic field. In (b) and (d) the wine (violet) curve is for Co magnetization along +y (-y) direction. The insets are the measurements set-up. There is no observable TAMR effect.

Supplementary Note 10. Additional data for samples I, II, III, and VIII

In the section, we show additional data for the samples presented in both the main text and section 6 of the supplementary information (tunneling contacts). Supplementary Figures 13-16 show additional data for sample I of the main text. Fig. 13 shows the non-local MR curves for different gate voltages, measured at 20 K. The black arrows represent the non-local MR values used for Figure 3 (a) of the main text. To ensure the signal comes from spin transport, in-plane Hanle curves for different gate voltages are measured in the parallel magnetization configuration (Fig. 14). This confirms the sign change in spin signal as a function of gate voltage. The data for gate voltage of -20 V are plotted separately in the inset because it possesses a large background drift unrelated to spin (the large drift is absent when antiparallel data are subtracted, as shown in Fig. 15). The Hanle curve for gate voltage of -10 V was not measured

because it exhibited no spin signal in the non-local MR. For quantitative analysis, the Hanle curves for parallel and antiparallel magnetizations are subtracted and the symmetric and antisymmetric components are separated (Fig. 15). As mentioned in section 7 above, the origin of the antisymmetric component is unknown but might be related to the edge geometry. More generally, it is not uncommon to have some antisymmetric component in graphene spin valves, and if such components exist then the symmetric component can be used to extract spin lifetimes. The key point here, however, is to show again that the polarity of the spin signal reverses with gate voltage, as can be seen by the symmetric component of the Hanle curves (blue curves in Fig. 15). These Hanle curves are subsequently fit to extract a spin signal amplitude (Fig. 16 (a)), spin lifetime (Fig. 16 (b)), and spin diffusion length (Fig. 16 (c)) as a function of gate voltage.

Samples II and III are the hybrid device that have both 1D edge contacts ('A' and 'B') as well as 2D contacts ('2D'). For sample II, Supplementary Figures 17, 18, and 19 show the non-local MR scans for 'A-2D' electrode pairs, 'B-2D' electrode pairs, and 'A-B' electrode pairs, respectively, for different gate voltages. The black arrows represent the non-local MR values used for Figure 4 (a) of the main text. For sample III, Supplementary Figures 20, 21, and 22 show the non-local MR scans for 'A-2D' electrode pairs, 'B-2D' electrode pairs, and 'A-B' electrode pairs, respectively, for different gate voltages. The black arrows represent the non-local MR values used for Figure 4 (b) of the main text.

As shown through the control measurements in sections 8 and 9, the observed signals are from spin transport as opposed to fringe field effects of TAMR. In a few scans, however, we have observed possible secondary contributions from fringe fields, as evidenced by a background of a single ferromagnet hysteresis loop in non-local MR measurement at -50 V, -40 V gate voltages shown in Supplementary Figure 21 and -20 V, 20 V, 30 V, 40 V gate voltages shown in Supplementary Figure 22.

Supplementary Figure 23 shows in-plane Hanle curves for several selected gate voltages measured in the parallel magnetization configuration to ensure the signals of samples II, III, and VIII come from spin transport. Supplementary Figures 23 (a)-23 (d) show Hanle curves for 'A-B' electrode pairs of sample II, 'B-2D' electrode pairs of sample III, 'A-B' electrode pairs of sample III and sample VIII (tunnel contacts in Supplementary section 6), respectively, at selected gate voltages.

Sample I

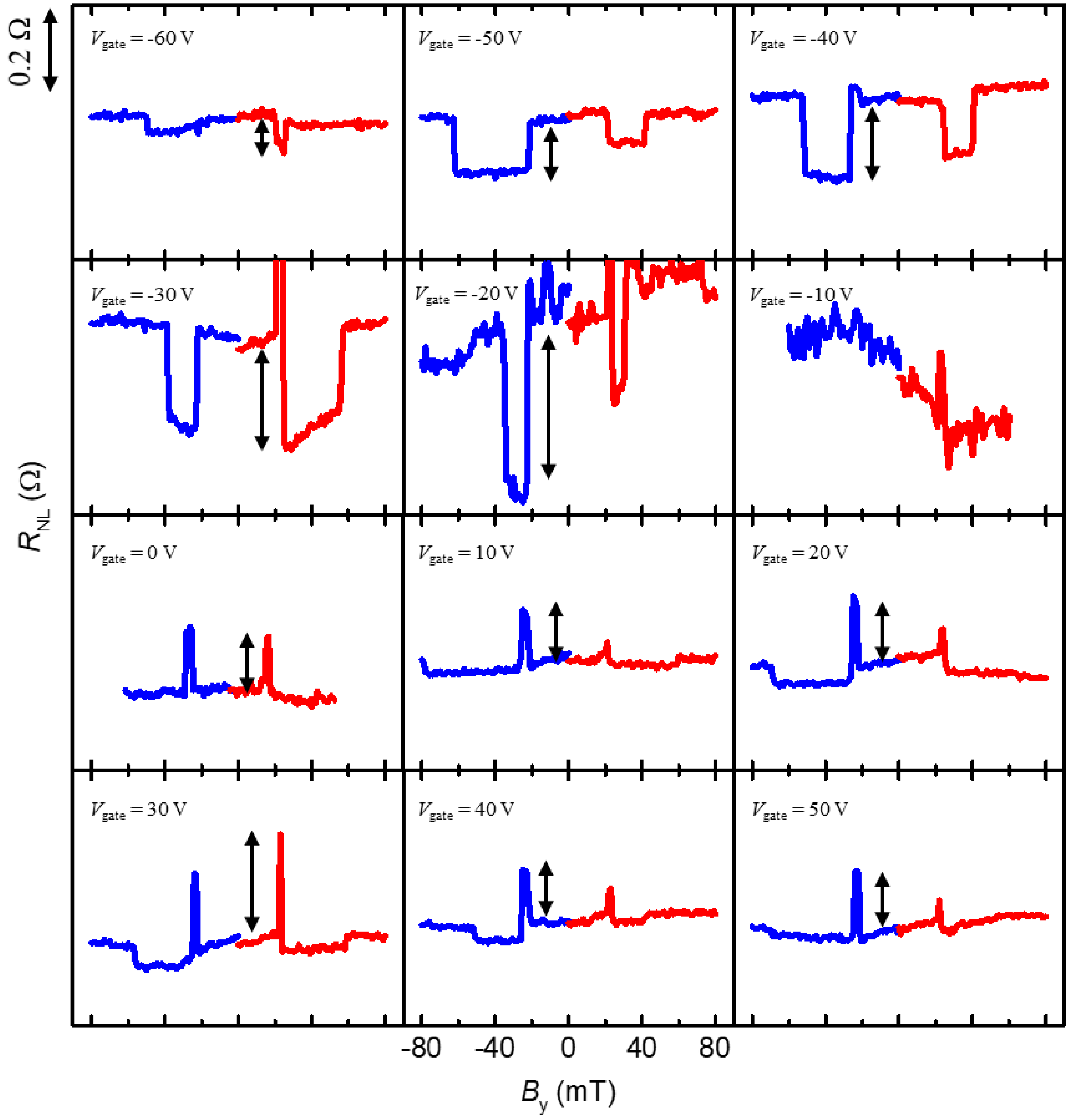

Supplementary Figure 13. Non-local MR curves at different gate voltage for Sample I, measured at 20 K. The red (blue) curve is for increasing (decreasing) magnetic field. For clarity, we only show the data after the magnetic field has passed zero value.

Sample I

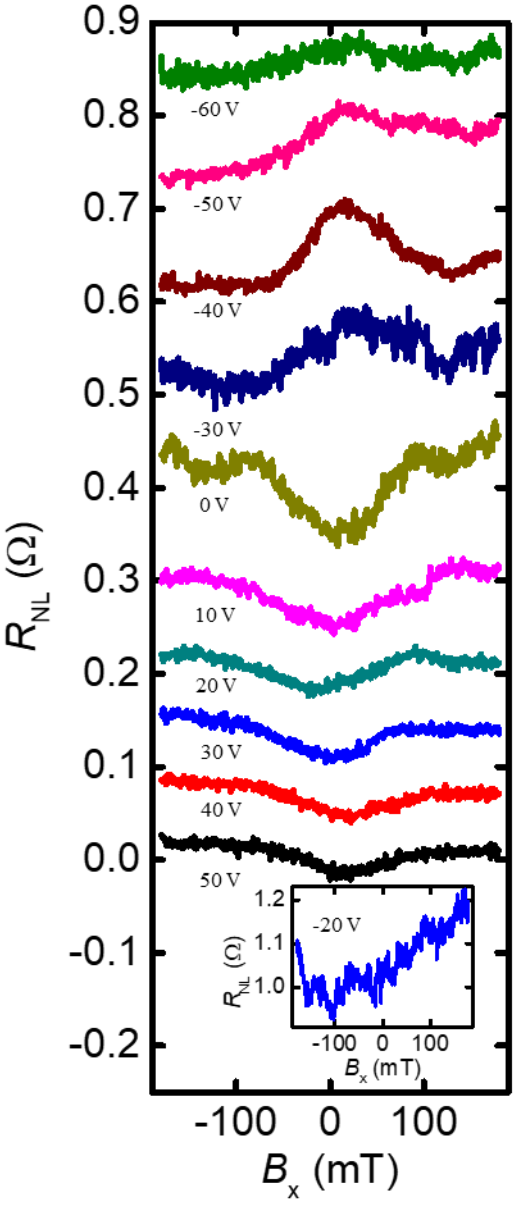

Supplementary Figure 14. Hanle curves when spin injector and spin detector magnetizations are parallel for Sample I at different gate voltage, measured at 20 K. The peak feature in the Hanle curve evolves into a dip with increasing gate voltage, confirming the sign change in non-local MR curves in Supplementary Figure 13. The Hanle data for V_{gate} = -20 V are plotted separately in the inset because of a large background signal unrelated to spin.

Sample I

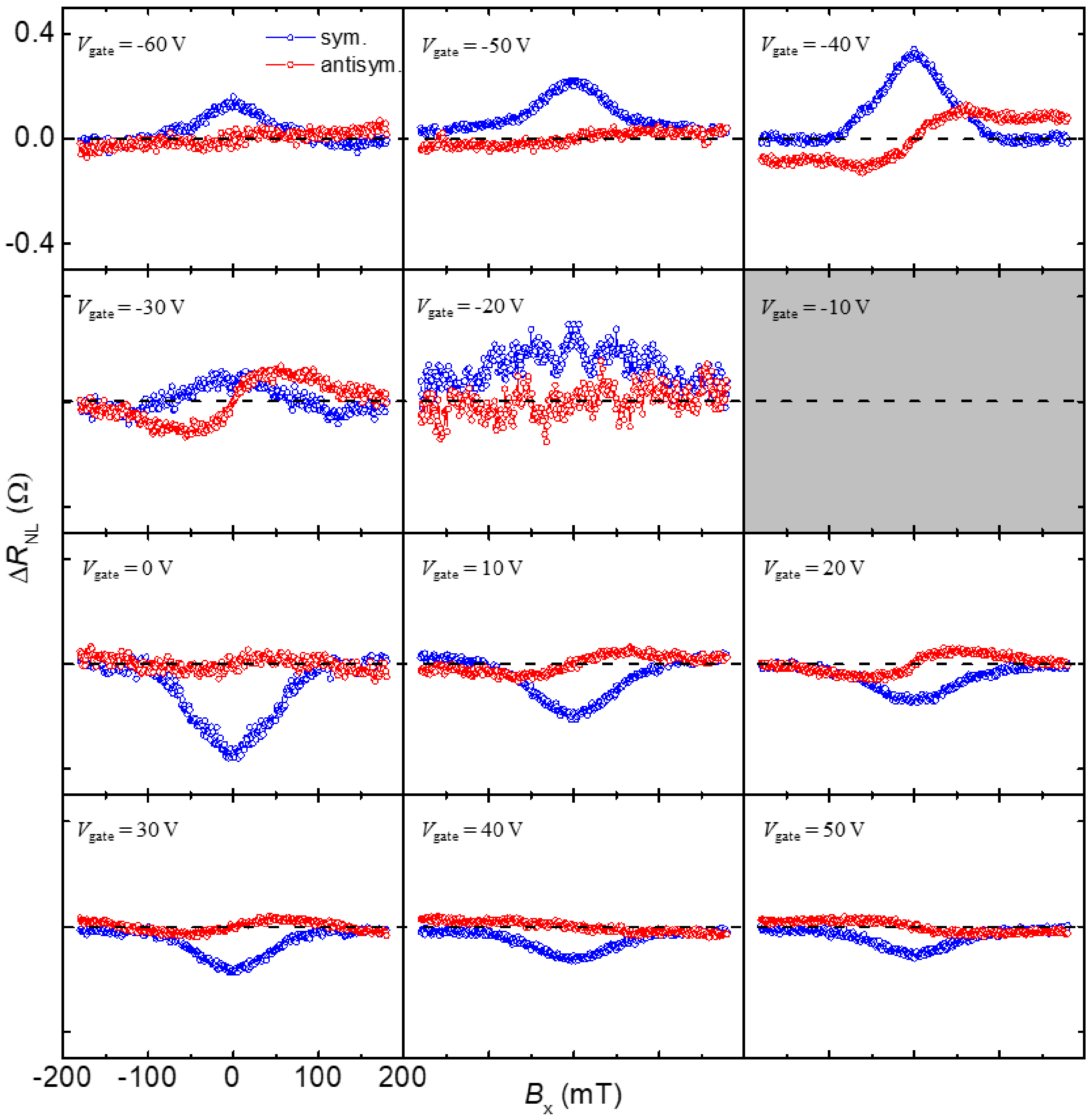

Supplementary Figure 15. The difference between the Hanle curves for parallel and antiparallel magnetization orientation at various gate voltages for Sample I, measured at 20 K. The blue (red) circles are for the symmetric (antisymmetric) component. With increasing gate voltage, the peak in the symmetric component (-60 V, -50 V, -40 V, -30 V, -20 V) evolves into a dip (0 V, 10 V, 20 V, 30 V, 40 V, 50 V), confirming the sign change in non-local MR curves in Supplementary Figure 13. All plots are on the same scale.

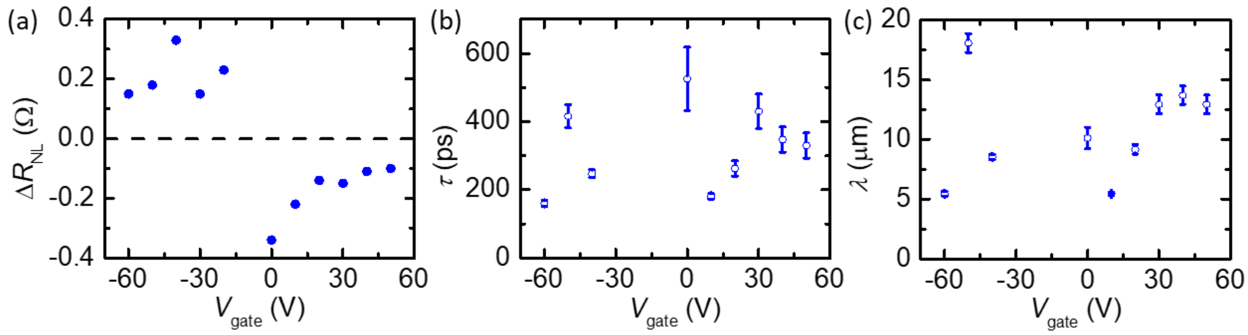

Supplementary Figure 16. (a) Spin signal magnitude extracted from the symmetric component of the Hanle curves in Supplementary Figure 15. (b) Spin lifetime and (c) Spin diffusion length of Sample I extracted by fitting the symmetric component of the Hanle curves in Supplementary Figure 15. Reliable Hanle fits could not be obtained for gate voltages of -20 V and -30 V.

Sample II electrode '2D' and 'A'

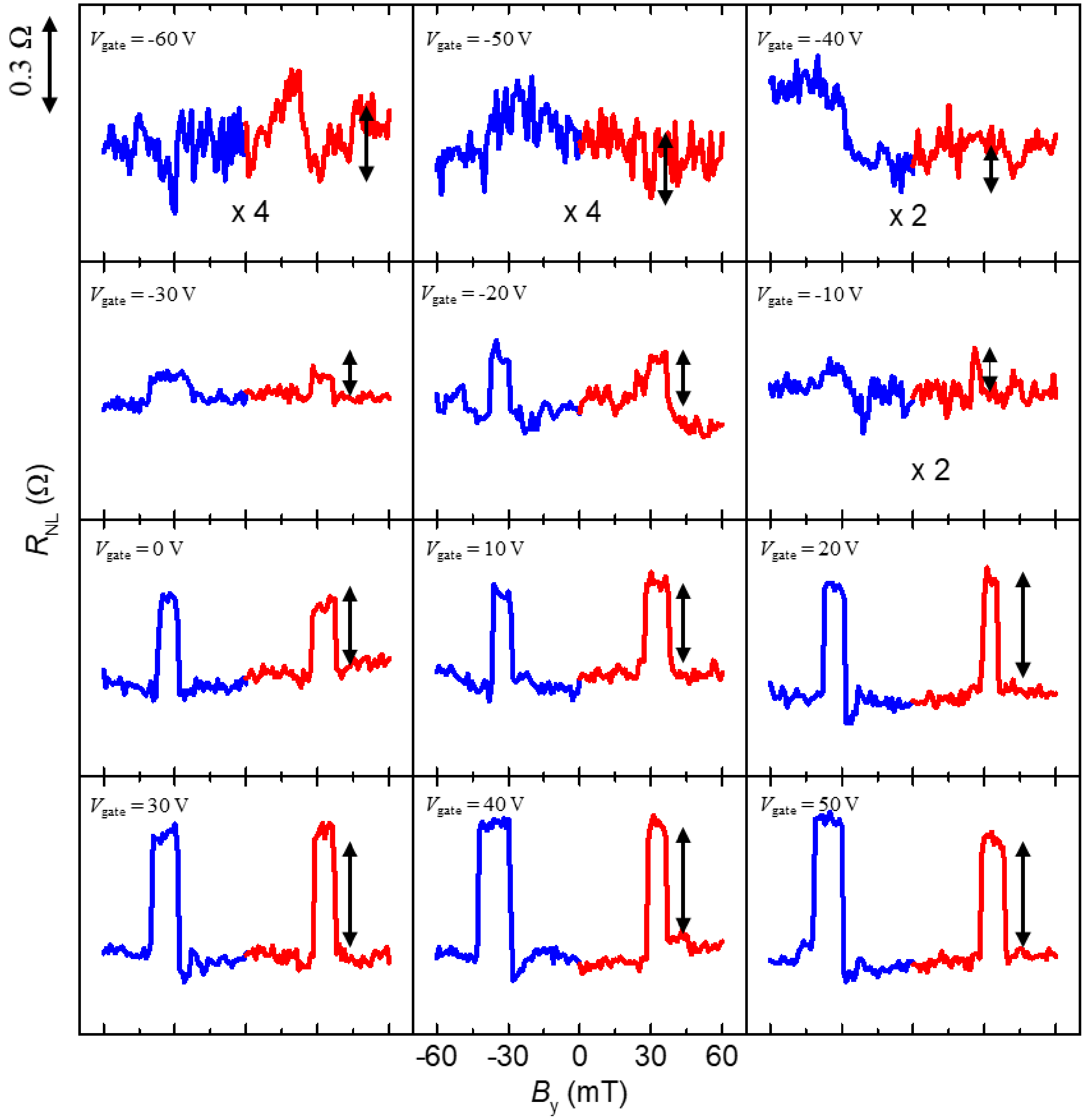

Supplementary Figure 17. Non-local MR curves between electrode '2D' and 'A' of Sample II (first row in main text Figure 4 (a)) at different gate voltages, measured at 20 K. The red (blue) curve is for increasing (decreasing) magnetic field. For clarity, we only show the data once the magnetic field passes zero value.

Sample II electrode ‘2D’ and ‘B’

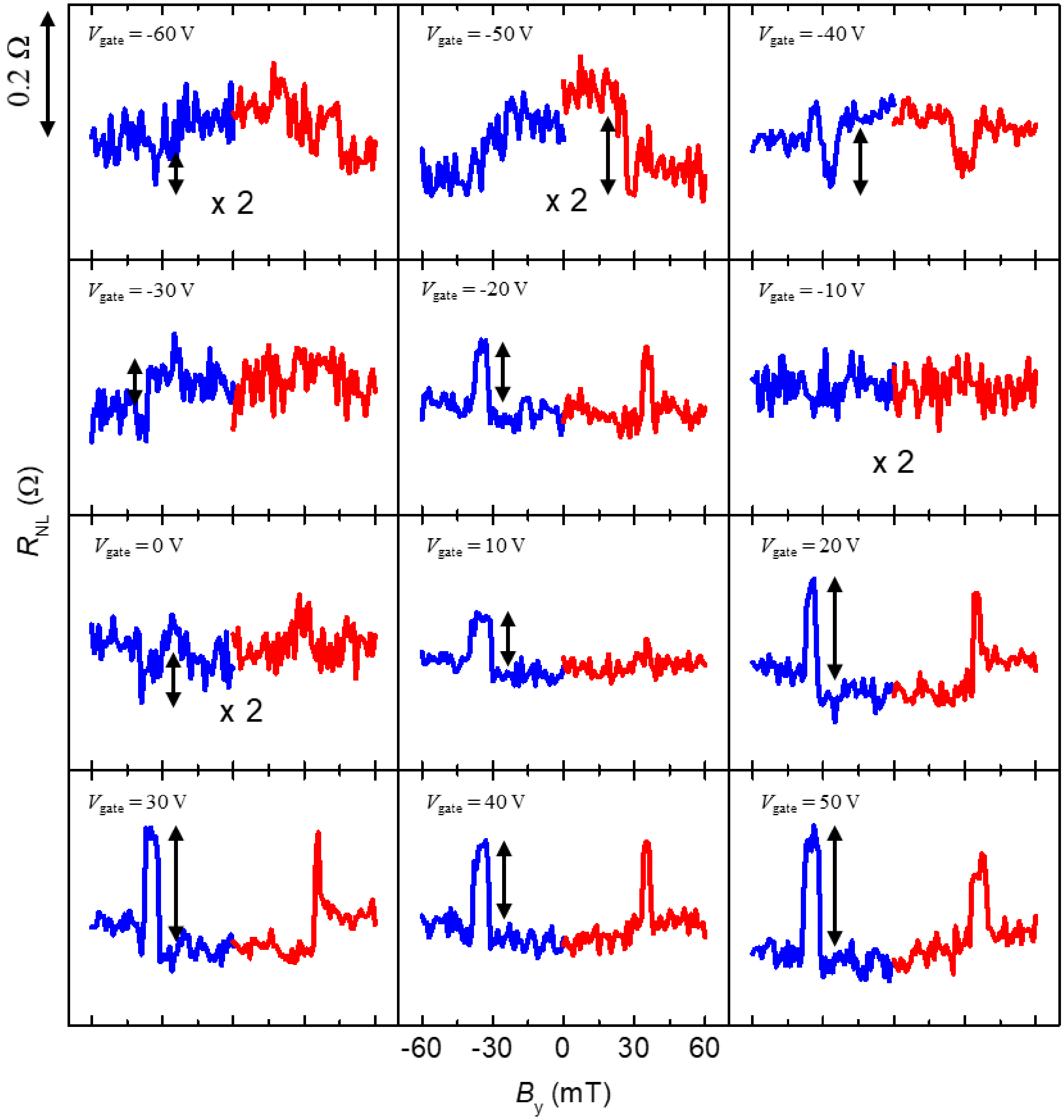

Supplementary Figure 18. Non-local MR curves between electrode ‘2D’ and ‘B’ of Sample II (second row in main text Figure 4 (a)) at different gate voltages, measured at 20 K. The red (blue) curve is for increasing (decreasing) magnetic field. For clarity, we only show the data once the magnetic field passes zero value.

Sample II electrode ‘A’ and ‘B’

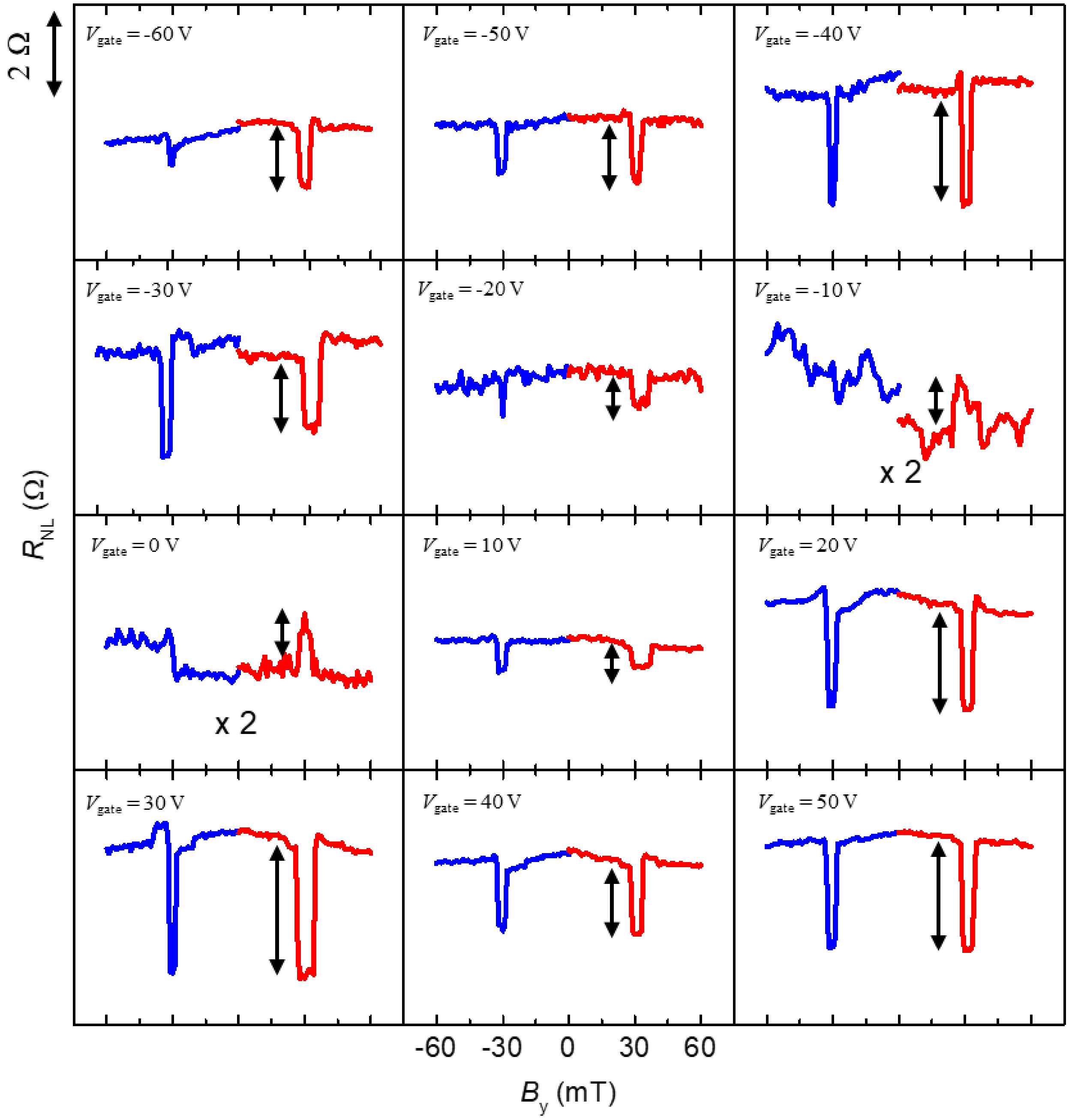

Supplementary Figure 19. Non-local MR curves between electrode ‘A’ and ‘B’ of Sample II (third row in main text Figure 4 (a)) at different gate voltages, measured at 20 K. The red (blue) curve is for increasing (decreasing) magnetic field. For clarity, we only show the data once the magnetic field passes zero value.

Sample III electrode '2D' and 'A'

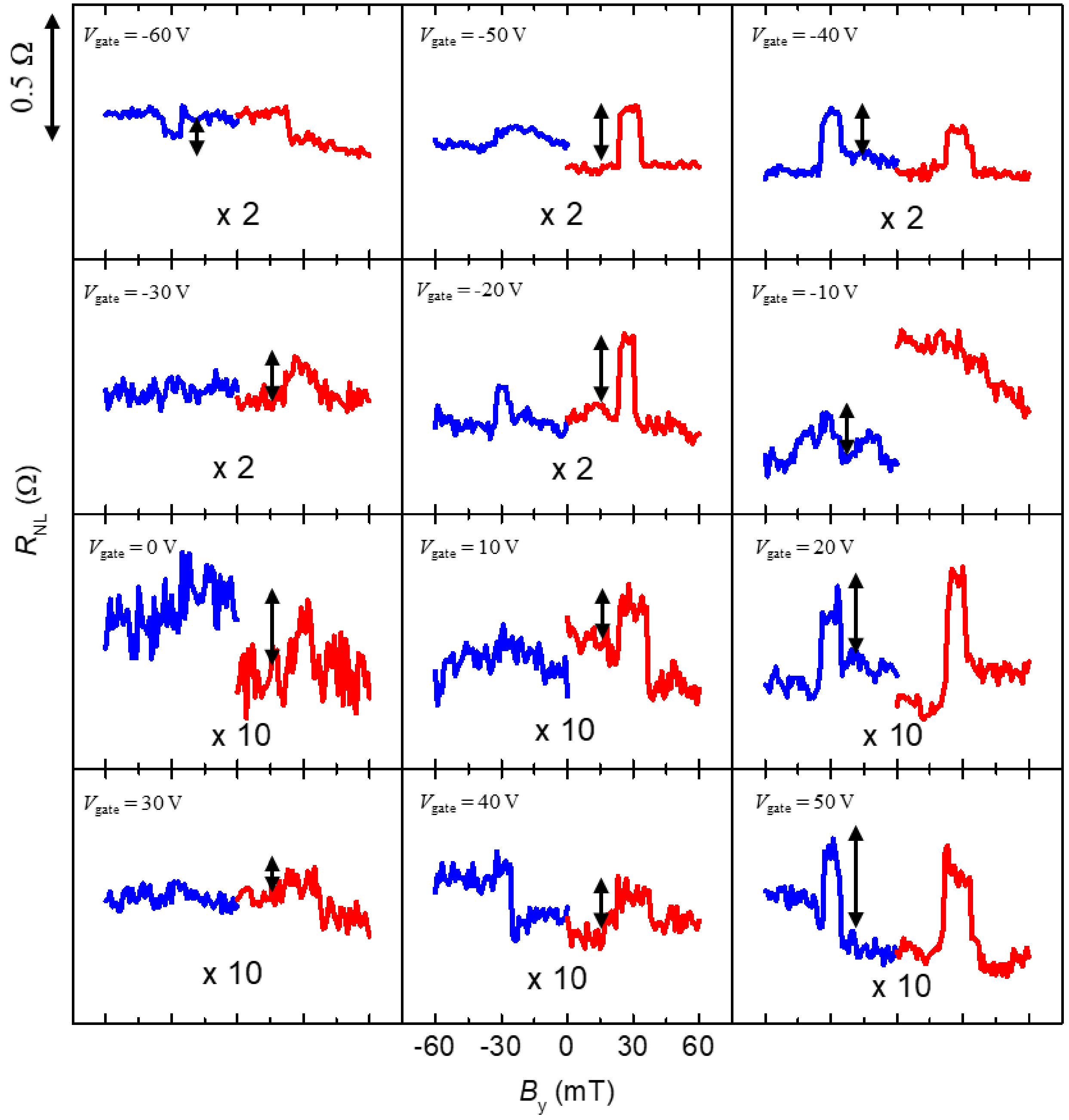

Supplementary Figure 20. Non-local MR curves between electrode '2D' and 'A' of Sample III (first row in main text Figure 4 (b)) at different gate voltages, measure at 20 K. The red (blue) curve is for increasing (decreasing) magnetic field. For clarity, we only show the data once the magnetic field passes zero value.

Sample III electrode ‘2D’ and ‘B’

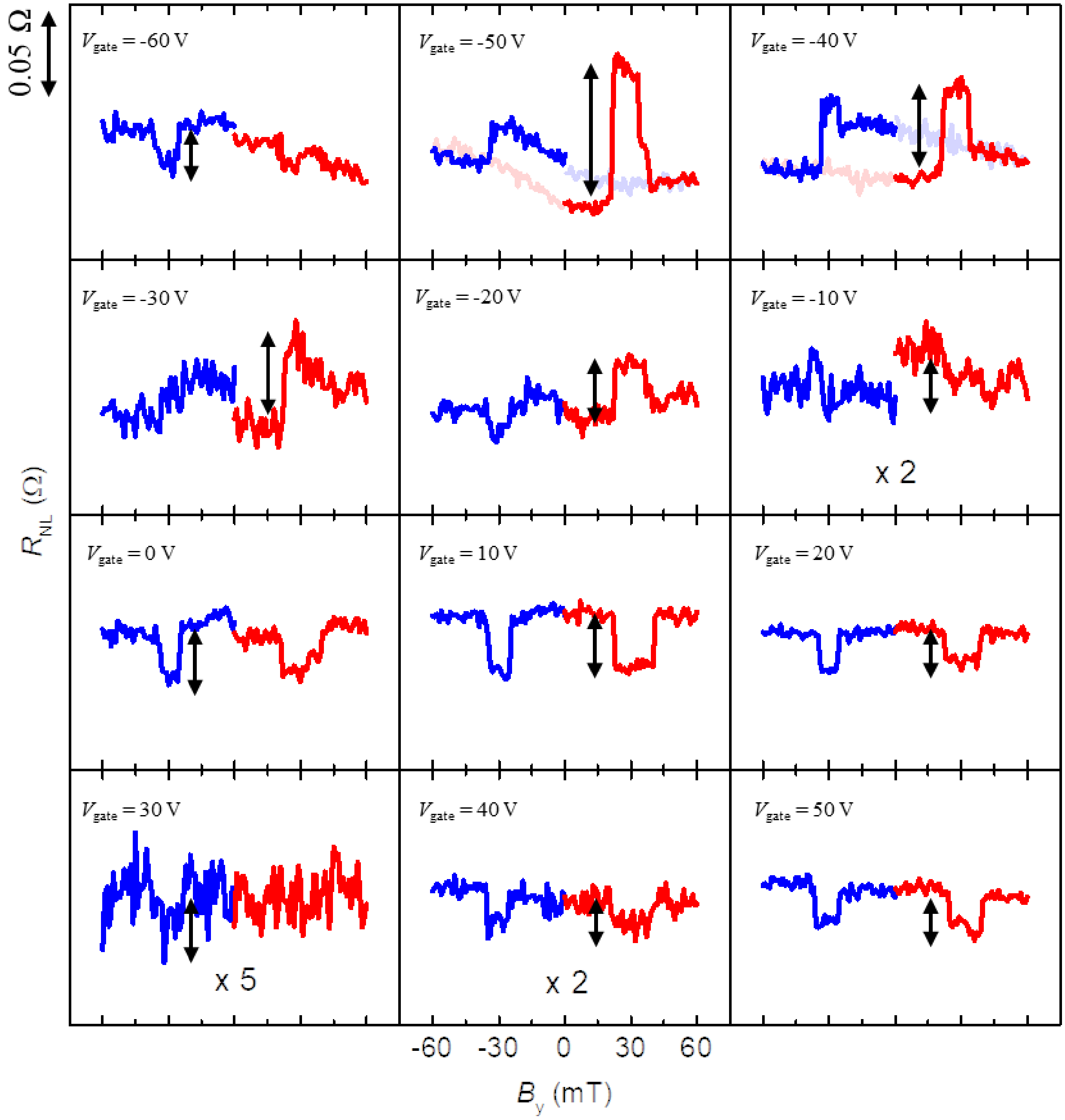

Supplementary Figure 21. Non-local MR curves between electrode ‘2D’ and ‘B’ of Sample III (second row in main text Figure 4 (b)) at different gate voltages, measured at 20 K. The red (blue) curve is for increasing (decreasing) magnetic field. For clarity, we only show the data once the magnetic field passes zero value for most gate voltage. For V_{gate} = -50 V and -40 V, we show data for the full sweep because of the observation of a ‘hysteresis loop’ like background, which could be due to some fringe field of the electrode.

Sample III electrode 'A' and 'B'

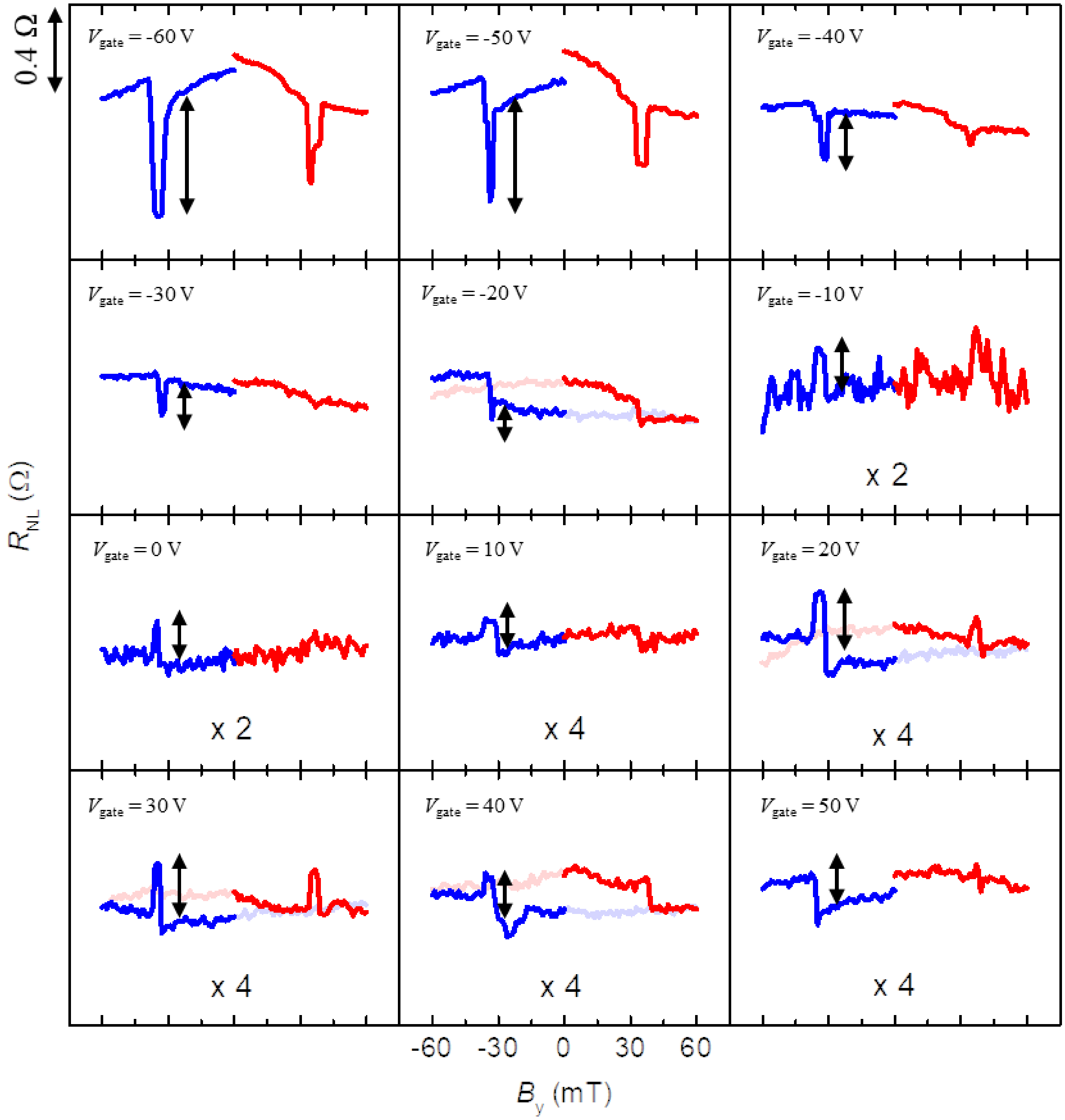

Supplementary Figure 22. Non-local MR curves between electrode 'A' and 'B' of Sample III (third row in main text Figure 4 (b)) at different gate voltages, measured at 20 K. The red (blue) curve is for increasing (decreasing) magnetic field. For clarity, we only show the data once the magnetic field passes zero value for most gate voltage. For V_{gate} = -20 V, 20 V, 30 V and 40 V, we show data for the full sweep because of the observation of a 'hysteresis loop' like background, which could be due to some fringe field of the electrode.

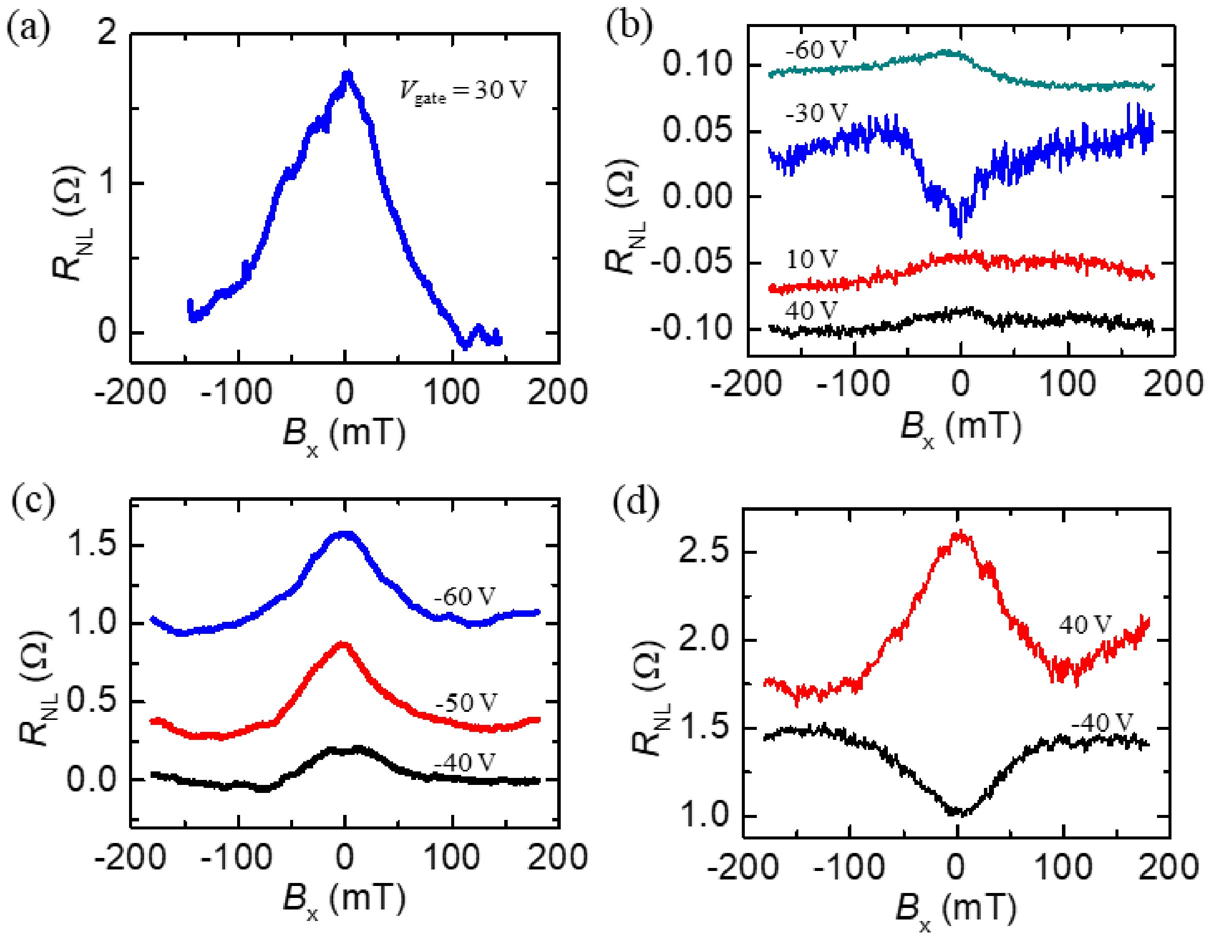

Supplementary Figure 23. Hanle measurement for confirming the spin transport of Sample II, III and VIII (spin injector and detector magnetizations are parallel). (a) Between electrode 'A' and 'B' of Sample II (third row in main text Figure 4 (a)). (b) Between electrode '2D' and 'B' of Sample III (second row in main text Figure 4 (b)). (c) Between electrode 'A' and 'B' of Sample III (third row in main text Figure 4 (b)). (d) Hanle of Sample VIII with tunneling barriers (Supplementary Figure 6).

1 Takahashi, S. & Maekawa, S. Spin injection and detection in magnetic nanostructures. *Phys. Rev. B* **67**, 052409 (2003).

2 Lazić, P., Belashchenko, K. D. & Žutić, I. Effective gating and tunable magnetic proximity effects in two-dimensional heterostructures. *Phys. Rev. B* **93**, 241401 (2016).

3 Sosenko, E., Wei, H. & Aji, V. Effect of contacts on spin lifetime measurements in graphene. *Phys. Rev. B* **89**, 245436 (2014).

4 Karpiak, B., Dankert, A., Cummings, A. W., Power, S. R., Roche, S. & Dash, S. P. 1D ferromagnetic edge contacts to 2D graphene/h-BN heterostructures. *2D Mater.* **5**, 014001 (2018).
